# T-cell Receptor Specificity Maintained by Altered Thermodynamics*[Fn FN2]

**DOI:** 10.1074/jbc.M113.464560

**Published:** 2013-05-22

**Authors:** Florian Madura, Pierre J. Rizkallah, Kim M. Miles, Christopher J. Holland, Anna M. Bulek, Anna Fuller, Andrea J. A. Schauenburg, John J. Miles, Nathaniel Liddy, Malkit Sami, Yi Li, Moushumi Hossain, Brian M. Baker, Bent K. Jakobsen, Andrew K. Sewell, David K. Cole

**Affiliations:** From the ‡ Cardiff University School of Medicine, Heath Park, Cardiff CF14 4XN, United Kingdom,; § Human Immunity Laboratory, Queensland Institute of Medical Research, Brisbane 4006, Australia,; ‖ Immunocore Ltd., Abingdon OX14 4RX, United Kingdom,; ¶ School of Medicine, University of Queensland, Brisbane 4006, Australia, and; the ** Department of Chemistry and Biochemistry, University of Notre Dame, Notre Dame, Indiana 46556

**Keywords:** Crystal Structure, Melanoma, Surface Plasmon Resonance (SPR), T-cell, T-cell Receptor, High Affinity T Cell Receptor (TCR), Peptide-major Histocompatibility Complex (pMHC)

## Abstract

The T-cell receptor (TCR) recognizes peptides bound to major histocompatibility molecules (MHC) and allows T-cells to interrogate the cellular proteome for internal anomalies from the cell surface. The TCR contacts both MHC and peptide in an interaction characterized by weak affinity (*K_D_* = 100 nm to 270 μm). We used phage-display to produce a melanoma-specific TCR (α24β17) with a 30,000-fold enhanced binding affinity (*K_D_* = 0.6 nm) to aid our exploration of the molecular mechanisms utilized to maintain peptide specificity. Remarkably, although the enhanced affinity was mediated primarily through new TCR-MHC contacts, α24β17 remained acutely sensitive to modifications at every position along the peptide backbone, mimicking the specificity of the wild type TCR. Thermodynamic analyses revealed an important role for solvation in directing peptide specificity. These findings advance our understanding of the molecular mechanisms that can govern the exquisite peptide specificity characteristic of TCR recognition.

## Teaser

**Background:** The molecular principles governing T-cell specificity are poorly understood.

**Results:** High affinity binding of a melanoma-specific T-cell receptor (TCR) is mediated through new MHC contacts and distinct thermodynamics.

**Conclusion:** A novel thermodynamic mechanism upholds TCR-peptide specificity.

**Significance:** TCRs can maintain peptide specificity using a mechanism that may enable widespread, safe enhancement of TCR binding affinity in therapeutic applications.

## Introduction

More than 30 different therapeutic monoclonal antibodies (mAbs) have Food and Drug Administration approval, and these soluble antigen receptors are being used in hundreds of current clinical trials for a wide range of disease states ranging from cardiovascular disease, cancer, and autoimmunity to induction of transplant tolerance (1, 2). Therapeutic application of the other class of antigen receptor, the T-cell receptor (TCR),^8^ has lagged behind the progress made with mAbs, but several recent studies have indicated that TCRs, or TCR/mAb hybrid molecules, might have a very bright future in gene therapy or as soluble molecules (3–5). TCRs have advantages over mAbs as they can exploit the MHC class I (MHCI) peptide presentation pathway to interrogate the internal proteome and thereby access a much wider range of disease targets than are available to mAbs. Antibodies undergo somatic hypermutation and bind with strong affinity (*K_D_* = nm–pm) and long half-lives (typically hours). In contrast, TCRs are only naturally expressed at the T-cell surface and bind foreign antigens with relatively weak affinities (*K_D_* = 100 nm to 270 μm) and short half-lives (0.1–12 s) (6, 7) with cancer-specific TCRs at the weaker end of this scale (7, 8). The weak affinity and short half-lives of natural TCR-pMHC interactions impose severe limitations on the therapeutic use of TCRs as soluble molecules. Recently, molecular engineering via phage display (5, 9, 10), yeast display (11), and computational design (12, 13) have provided a route to circumvent the intrinsic weak binding affinity of TCRs. Just a few mutations within the TCR CDR loops can improve the binding affinity of a TCR to antibody-like levels and beyond (9–11). These developments have paved the way for the use of enhanced TCRs as soluble therapies. Indeed, we have recently shown that high affinity soluble “monoclonal” TCRs can be used to target cancer antigens at the cell surface and induce tumor regression (14), and this approach is now being trialed at several centers.

Despite the promise of affinity-enhanced TCRs, concerns remain about their peptide specificity (15, 16). Complete immune cover requires that a limited number of TCRs are able to recognize the vastly greater number of potential foreign peptides that could be encountered (17, 18). As a result, TCRs are said to be “cross-reactive” or “poly-specific” (17, 19). The ability of individual TCRs to recognize huge numbers of peptides has raised significant concerns with regard to TCRs that have undergone artificial affinity enhancement *in vitro*. These reservations stem from the possibility that high affinity TCRs, particularly those where interaction between the TCR and the MHC component have been increased, might recognize an even greater number of cognate peptides. As enhanced TCRs have not undergone thymic selection *in vivo*, there are concerns that increased peptide cross-recognition by enhanced affinity TCRs might extend to the binding of self-peptides with a high enough affinity to induce off-target pathology. To date, there has been no rigorous testing of the specificity of an enhanced affinity TCR using molecular approaches.

Here we explored these issues using phage display to generate a high affinity TCR derived from the MEL5 TCR specific for the heteroclitic version of the HLA-A*0201-restricted primary melanoma antigen recognized by T-cells 1 (MART-1_26–35_) peptide, ELAGIGILTV (A2-ELA) (10, 20). This TCR, α24β17, bound to A2-ELA with an affinity 30,000 times stronger than the natural parent MEL5 TCR, resulting in picomolar levels of binding (10). Comparison of the crystallographic structure of the α24β17 TCR and wild type MEL5 TCR (21) in complex with A2-ELA showed that this remarkable enhancement in binding was attributable to new or altered contacts with the MHC protein. Surprisingly though, α24β17 remained exquisitely peptide-specific, mimicking the specificity of the wild type receptor. Structural and thermodynamic investigations highlighted the role of solvent in determining peptide specificity, a novel finding that sheds light on the molecular rules that govern TCR specificity in general. Overall, we provide a new molecular mechanism by which TCRs maintain peptide specificity and show that it is possible to affinity mature TCRs for therapeutic use as soluble molecules without concomitant loss of peptide specificity.

## EXPERIMENTAL PROCEDURES

### 

#### 

##### Phage Display Selection of the High Affinity TCR, α24β17

Construction of the vector for displaying the wild type MEL5 TCR on the surface of phage was identical to previous reports (10). The variable region of the TCR α (residues 25–100) and β (residues 25–106) chains (including the CDR1, -2, and -3 loops) was targeted for introducing mutations using primers that were specific for the beginning of the TCR variable domain for each chain. Mutations were introduced as previously described (10). High affinity MEL5-derived TCRs were selected by panning the phage libraries on immobilized HLA-A2-ELAGIGILTV as previously reported (10), and the strongest binders were selected for additional rounds of phage display. Thus, the α24β17 TCR was selected based only on its ability to bind with high affinity to HLA-A2-ELAGIGILTV.

##### Generation of Expression Plasmids

The α24β17 TCR α and β chains, HLA-A*0201 heavy chain and β2m chain were generated by PCR mutagenesis (Stratagene) and PCR cloning. All sequences were confirmed by automated DNA sequencing (Lark Technologies). The TCR sequences were constructed implementing a disulfide-linked construct to produce the soluble domains (variable and constant) for both the α (residues 1–207) and β chains (residues 1–247) (22, 23). The HLA-A*0201 heavy chain (residues 1–248) (α1, α2, and α3 domains), tagged or not tagged with a biotinylation sequence, and β2m (residues 1–100) were also cloned and used to make the pMHCI complexes. The TCR α and β chains, the HLA-A*0201 α chain and β2m sequences were inserted into separate pGMT7 expression plasmids under the control of the T7 promoter (22).

##### Protein Expression, Refolding, and Purification

Competent Rosetta DE3 *E. coli* cells were used to produce the TCR α and β chains, HLA-A*0201 heavy chain, and β2m in the form of inclusion bodies using 0.5 mm isopropyl 1-thio-β-d-galactopyranoside to induce expression as described previously (22, 24, 25).

##### pMHCI Biotinylation

Biotinylated pMHCI was prepared as previously described (26).

##### Surface Plasmon Resonance (SPR) Experiments

SPR Equilibrium binding analysis was performed using a BIAcore T100^TM^ equipped with a CM5 sensor chip as previously reported (26–28). HLA-DR1, generated as in Cole *et al.* (29), was used as a negative control on flow cell 1. SPR kinetic analyses were carried out to determine the *K_D_* values for the TCR at 25 °C. For all kinetic experiments, ∼300 response units of pMHC were coupled to the CM5 sensor chip surface. The TCR was then injected at concentrations ranging from 10× above and 10× below the known *K_D_* of the interaction at 45 μl/min. The *K_D_* values were calculated assuming 1:1 Langmuir binding (AB = B × AB_MAX_/(*K_D_* + B)), and the data were analyzed using a global fit algorithm (BIAevaluation^TM^ 3.1). SPR kinetic titration analysis was used to examine the binding of the α24β17 TCR across a greater range of concentrations. The TCR was analyzed at five concentrations that represented the maximum range that could be accurately achieved around the *K_D_* of the interaction. During the analysis, ∼200 response units of pMHC were immobilized onto the CM5 sensor chip surface. Each concentration of TCR was injected at a high flow rate of 45 μl/min for a 240-s association period and a 120-s dissociation period. The final and highest concentration had a longer dissociation period of 3600 s. A fast flow rate and a low amount of immobilized pMHC were used to limit the association and dissociation mass transfer limitations as recommended by the experts at BIAcore^TM^. The *k*_on_, *k*_off_, and *K_D_* values were calculated by global fitting of the data using BIAevaluation^TM^ 3.1 software and the single-cycle kinetics method (30). For the thermodynamics experiments we used the *K_D_* determined by SPR at different temperatures with the standard thermodynamic equation Δ*G* = *RT*ln*K_D_* and the standard non-linear Van't Hoff equation (Δ*G*° = Δ*H*° − *T*Δ*S*° + ΔCp°(*T* − *T*_0_) − *T*ΔCp° ln(*T*/*T*_0_)) with *T*_0_ = 298 K.

##### Crystallization, Diffraction Data Collection, and Model Refinement

All protein crystals were grown at 18 °C by vapor diffusion via the sitting drop technique. 200 nl of 1:1 molar ratio TCR and pMHCI (10 mg/ml) in crystallization buffer (10 mm Tris, pH 8.1, and 10 mm NaCl) was added to 200 nl of reservoir solution. α24β17 crystals were grown using our in-house TCR/pMHC optimized protein crystallization screen (TOPS) in 0.1 m sodium cacodylate, pH 6.5, 20% PEG 8000, and 0.2 m ammonium sulfate (31); α24β17-A2-ELA crystals were grown in TOPS4 in 0.1 m HEPES, pH 7.0, 20% PEG 4000, and 0.2 m ammonium sulfate (31); α24β17-A2-ELA4A crystals were grown in TOPS3 in 0.1 m Tris, pH 7.5, 15% PEG 4000, and 17.4% glycerol (31); α24β17-A2-ELA7A crystals were grown in TOPS4 in 0.1 m Bistris propane, pH 7.0, 20% PEG 4000, 0.2 m ammonium sulfate, and 17.4% glycerol (31); A2-ELA1A crystals were grown in 25 mm MES, pH 6.5, 24% PEG 3350, and 10 mm NaCl; A2-ELA4A crystals were grown in TOPS in 0.1 m sodium cacodylate, pH 6.0, 20% PEG 8000, and 0.2 m ammonium sulfate (31); A2-ELA8A crystals were grown in 25 mm MES, pH 6.5, 24% PEG 3350, and 10 mm NaCl. All crystals were soaked in 30% ethylene glycol before cryo-cooling. All crystallization screens and optimization experiments were completed using an Art-Robbins Phoenix dispensing robot (Alpha Biotech Ltd.). Data were collected at 100 K at the Diamond Light Source (DLS), Oxfordshire, UK or the Advanced Photon Source at Argonne National Laboratory. All datasets were collected at a wavelength of 0.98 Å using an ADSC Q315 CCD detector. Reflection intensities were estimated with the XIA2 package (32), and the data were scaled, reduced, and analyzed with SCALA and the CCP4 package (33). Structures were solved with molecular replacement using PHASER (34). Sequences were adjusted with COOT (35), and the models were refined with REFMAC5. Graphic representations were prepared with PyMOL (36). The reflection data and final model coordinates were deposited with the PDB database (α24β17, PDB 4JFH; α24β17-A2-ELA, PDB 4JFF; α24β17-A2-ELA4A, PDB 4JFD; α24β17-A2-ELA7A, PDB 4JFE; A2-ELA1A, PDB 4JFO; A2-ELA4A, PDB 4JFP; A2-ELA8, PDB 4JFQ).

## RESULTS

### 

#### 

##### The α24β17 TCR Binds to A2-ELA with High Affinity Due to an Extended Off-rate

**FIGURE 1.**
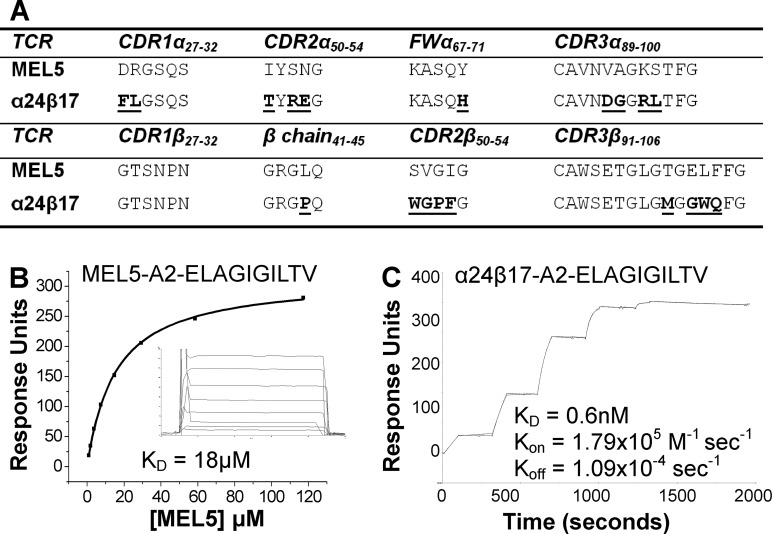
**Sequence and kinetic analysis of the HLA A2-ELAGIGILTV specific high affinity TCR, α24β17.** *A*, shown is sequence alignment of the α24β17 TCR and the wild type MEL5 TCR progenitor. Mutations from the MEL5 TCR wild type sequence are in *bold* and *underlined. B* and *C*, these data were produced on a BIAcore T100^TM^ using SPR and then analyzed using equilibrium analysis (*B*) and kinetic titration analysis (*C*). Kinetic titration analysis is an improved method for analyzing the kinetic parameters of high affinity interactions with long off-rates. Each TCR was analyzed at five concentrations that represented the greatest range we could accurately achieve around the *K_D_* of each interaction, and the data were analyzed using the kinetic titration analysis algorithm (BIAevaluation^TM^ 3.1) (30). The raw SPR data and the fits are shown in each panel. These data illustrate the improved binding capabilities of the HLA A2-ELAGIGILTV-specific high affinity mutant TCR, α24β17 (*C*). The α24β17 TCR bound to HLA A2-ELAGIGILTV with 30,000 times stronger affinity compared to the MEL5 TCR.

To generate a high affinity version of the MEL5 TCR, we implemented phage display as previously described (10). This process produced a high affinity TCR, α24β17, that varied from the MEL5 TCR parental sequence at 19 amino acids located within the CDR1α, CDR2α, FWα, CDR3α, CDR2β, and CDR3β loops as well as in the β chain between residues 41 and 45 (Fig. 1*A*). We previously demonstrated that the wild type MEL5 TCR binds to A2-ELA with a dissociation constant (*K_D_*) of 18 μm (7, 20, 21) (Fig. 1*B*). The high affinity α24β17 TCR bound to A2-ELA with 30,000-fold stronger affinity (*K_D_* = 0.6 nm) (Fig. 1*C*) due primarily to a slower off-rate of 1.09 × 10^−4^ s^−1^. The off-rate for the MEL5 TCR was too fast to measure (>0.1 s^−1^) (Fig. 1*B*).

##### The MEL5 and α24β17 TCRs Use a Similar Binding Mode to Engage A2-ELA

**FIGURE 2.**
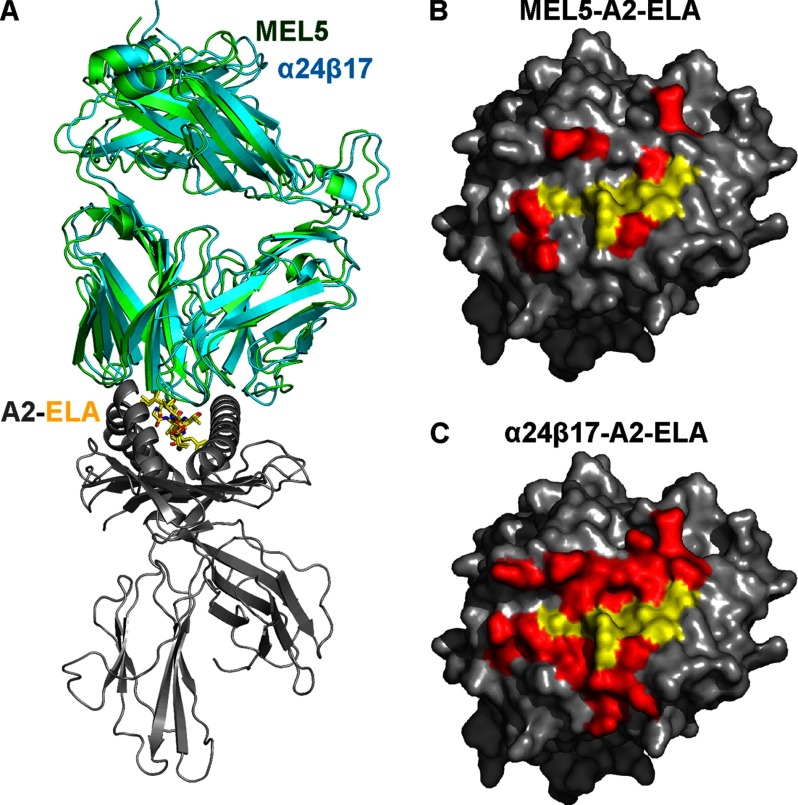
**Structural analysis of the binding mode implemented by MEL5 *versus* α24β17 when interacting with A2-ELA.** *A*, shown is the overall binding mode of MEL5 (data are from our previously published work (21)) (*green schematic*) and α24β17 (*cyan schematic*) interaction with HLA-A*0201 (*gray schematic*) and the ELAGIGILTV peptide (*yellow sticks*). Generally, the two TCRs bind in a very similar orientation, with some differences in the CDR loops and more flexible regions of the TCR variable and constant domains. *B* and *C*, a surface representation of the A2-ELA complex looking down at the peptide is shown. MHC residues that are contacted by the TCR are colored in *red*. Peptide residues that are contacted by the TCR are colored *yellow*. From this analysis, it is clear that although MEL5 (*B*) and α24β17 (*C*) make a similar contact footprint with the peptide (*yellow*), α24β17 makes substantially more interactions with the MHC surface (*red*).

Previous structures of high affinity TCRs produced by phage display have shown that, although these mutated TCRs can bind with many orders of magnitude stronger affinity than their wild type progenitors, they bind with a similar overall conformation (9, 37). This observation is important because this conserved binding mode increases the likelihood that high affinity-modified TCRs can maintain the rules that govern T-cell antigen recognition and self-tolerance. To determine the structural basis of high affinity binding for the α24β17 TCR, we solved the α24β17-A2-ELA complex structure to 2.4 Å. Molecular replacement was successful only in space group P4_1_, and the resolution was sufficiently high to show that the interface between the two molecules was well ordered and contained well defined electron density (supplemental Fig. S1). The crystallographic *R*_work_/*R*_free_ factors were 21% and 26.3%, respectively. The ratio was within the accepted limits shown in the theoretically expected distribution (38) (supplemental Table S1). The overall buried surface area (BSA) of 2705 Å^2^ (TCR-pMHC) for α24β17-A2-ELA was slightly higher than that for our previously published structure of MEL5-A2-ELA (21) (BSA, 2327.8 Å^2^) (Table 1) but was within the observed range for natural TCR-pMHC interactions (39). The high affinity α24β17 TCR bound with a diagonal docking geometry to A2-ELA as previously reported for other TCR-pMHC complexes (Fig. 2*A*) (39). We observed a high level of similarity between the MEL5-A2-ELA and α24β17-A2-ELA complexes, suggesting that the overall conformation was not substantially affected by the mutations in α24β17. The crossing angle of both TCRs (48° for MEL5 and 42° for α24β17) (supplemental Fig. S2*A*) was similar and fell within the previously observed range for TCR-pMHC complexes (39). The positioning of the complementarity-determining region (CDR)-loops over the A2-ELA surface was also similar for both TCRs (supplemental Fig. S2*B*). Importantly, the ELA peptide conformation was virtually identical in both complexes, discounting the possibility that changes in peptide structure contributed to the affinity enhancement (supplemental Fig. 2, *C* and *D*). Thus, differences in binding affinity between the MEL5 and α24β17 TCRs could not be explained by a large conformational change in geometry, in agreement with our previously published observations (9, 37).

**TABLE 1:** **Summary of co-complex structures of α24β17-A2-ELA, α24β17-A2-ELA4A, and α24β17-A2-ELA7A**

Parameter	MEL5-A2-ELA	α24β17-A2-ELA	α24β17-A2-ELA4A	α24β17-A2-ELA7A
H-bonds (≤3.2 Å)	6	16	15	16
H-bonds (≤3.4 Å)	1	6	6	1
vdW (≤3.5 Å)	23	21	11	15
vdW (≤4 Å)	65	95	109	105
Total contacts	95	138	141	136
CDR1/CDR2/CDR3 contacts (≤4Å)				
α chain	26/11/13	37/12/21	38/16/18	31/8/20
β chain	3/6/36	1/38/30	1/41/27	1/46/30
Peptide contacts	39	42	39	41
MHC contacts	56	97	102	95
Crossing angle	42°	48°	48°	48°
Buried surface area (Å^2^)	2327.8	2705	2650.4	2781.6
Surface complementarity				
TCR-MHC	0.55	0.71	0.65	0.66
TCR-peptide	0.71	0.59	0.58	0.62
TCR-pMHC	0.6	0.66	0.62	0.63

##### Increased TCR-MHC Interactions Underlie the High Affinity Binding of the α24β17 TCR

**FIGURE 3.**
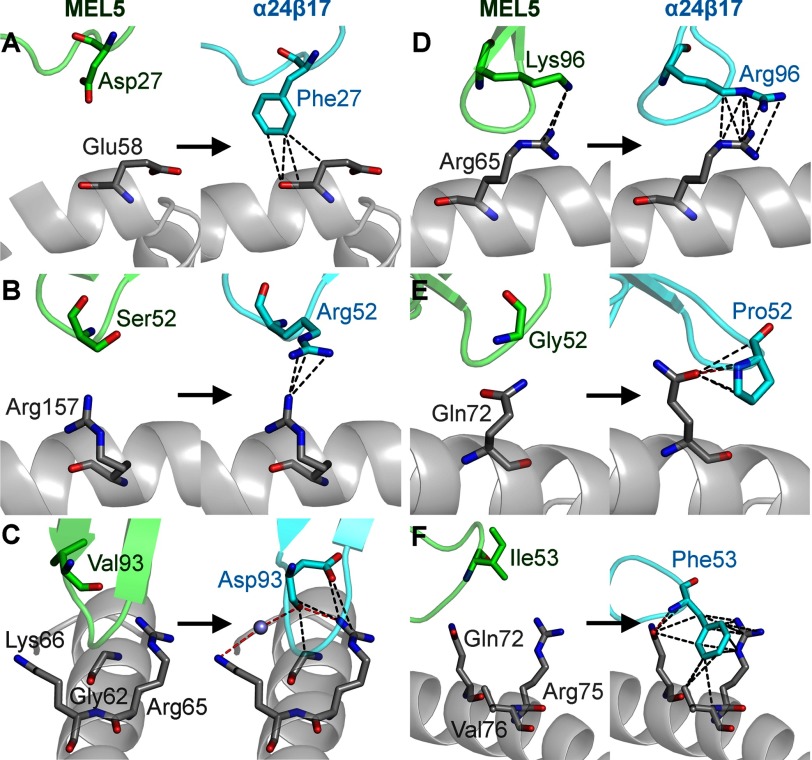
**The α24β17-mutated residues make an increased number of contacts with the MHC surface compared with MEL5 wild type residues.** MEL5 residues are shown on the *left-hand side of each panel in green*. The equivalent α24β17 residues are shown on the *right-hand side of each panel in cyan*. Hydrogen bonds (<3.4 Å) are shown as *red dotted lines* and van der Waals interactions (<4.0 Å) are shown as *black dotted lines*. In all cases an increased number of interactions was observed between α24β17 and the MHC surface compared with MEL5. Shown are position 27 in the TCR CDR1α loop (*A*), position 52 in the TCR CDR2α loop (*B*), position 93 in the TCR CDR3α loop (*C*), position 96 in the TCR CDR3α loop (*D*), position 52 in the TCR CDR2β loop (*E*), and position 53 in the TCR CDR2β loop (*F*).

Previous structures of high affinity TCRs have shown that just a small number of additional contacts at the binding interface can mediate huge improvements in TCR binding affinity (9, 37). To delineate the mechanism behind the high affinity binding of the α24β17 TCR, we investigated the binding interface in atomic detail. Although the overall conformations of the MEL5 and α24β17 TCRs in complex with A2-ELA were comparable, there were a number of important interfacial differences that could explain the divergent binding affinities. The interactions with the ELAGIGILTV peptide were very similar for MEL5 and α24β17 TCRs, making 39 and 42 contacts, respectively (Table 1). However, the α24β17 TCR utilized a substantial number of new contacts with the MHC surface, making 97 MHC contacts compared with only 56 for MEL5 (Table 1, Fig. 2, *B* and *C*). This increase in interactions with the MHC was consistent with the increased BSA for α24β17-A2-ELA (Table 1) and was probably the main mechanism enabling α24β17 to bind with an affinity 30,000-fold stronger than MEL5 to A2-ELA. The majority of the new contacts were directly attributable to the mutated residues in the α24β17 TCR. For instance, MEL5 TCRα chain residue Asp-27 made no contacts with the MHC surface. However, when mutated in the α24β17 TCRα chain, the longer aromatic side chain of residue Phe-27 was able to make four vdW contacts with MHC residue Glu-58 (supplemental Table S2, Fig. 3*A*). Similarly, the mutation from MEL5 TCRα chain residue Val-93 to Asp-93 in the α24β17 TCRα chain resulted in four new vdW contacts and two new hydrogen bonds, and the mutation from MEL5 TCRβ chain residue Ile-53 to Phe-53 in the α24β17 TCRβ chain resulted in 18 new vdW contacts and 1 new hydrogen bond (supplemental Table S2, Fig. 3). Overall, mutated residues in the α24β17 TCR accounted for 36 new vdW contacts and 3 new hydrogen bonds with the MHC surface (supplemental Table S2). Thus, the vast majority of the 44 new contacts formed by the α24β17 TCR were made directly by mutated residues with only a small number of new contacts made through indirect effects of the high affinity mutations on non-mutated residues (Table 1, supplemental Table S3). Our observation, that the α24β17 TCR mediated enhanced affinity through an increase in MHC contacts, substantially altered the ratio of TCR-peptide contacts *versus* TCR-MHC contacts compared with MEL5. For instance, the MEL5 TCR made 41% of the total contacts with the peptide, compared with just 30% for the α24β17 TCR. This change in focus, from peptide interactions to MHC interactions, raised the possibility that the α24β17 TCR might bind in a peptide-independent manner. To investigate this possibility further, we probed the ability of α24β17 to tolerate substitutions in the ELAGIGILTV peptide.

##### The α24β17 TCR Remains Highly Sensitive to Peptide Substitutions

The α24β17-A2-ELA structure demonstrated that new MHC contacts were central to the 30,000-fold stronger affinity compared with the MEL5-A2-ELA complex. This enhancement did not enable α24β17 to bind to other HLA-A2-restricted peptides that were used as negative controls in different SPR experiments, including A2-ILAKFLHWL, A2-SLLMWITQC, and A2-YLEPGPVTA (data not shown). We then investigated the ability of α24β17 to tolerate changes in the cognate ELAGIGILTV peptide by performing an alanine mutagenesis scan across the peptide backbone and evaluating the capacity of the A2-ELA mutants to bind α24β17 using SPR (Table 2, supplemental Fig. S3). As peptide positions P2 and P10 were buried and are known to be important for MHC binding (40), we focused on assessing the adjacent residues at positions P1, P4, P5, P6, P7, P8, and P9. We omitted P3, as this residue is already Ala in the native sequence. Strikingly, modification of any peptide residue to Ala reduced the binding of α24β17 to wild type-like affinities or lower (Table 2). For example, α24β17 bound A2-ELA with Gly substituted for Ala at position 4 (A2-ELA4A) with a *K_D_* of 36 μm, 60,000-fold weaker affinity than for A2-ELA (ΔΔ*G*° = 6.51 kcal/mol) (Table 2). In the α24β17-A2-ELA structure, four hydrogen bonds and eight vdW contacts were made between the TCR and peptide residue Gly-4 (supplemental Table S3). Thus, although Ala and Gly are similar in terms of size and charge, this mutation could potentially result in the disruption of an important network of TCR-peptide contacts. Similarly, α24β17 bound A2-ELA with Ile substituted for Ala at position 7 (A2-ELA7A), which resulted in a *K_D_* of 31 μm, >50,000-fold weaker affinity than for A2-ELA (ΔΔ*G*° = 6.42 kcal/mol) (Table 2). The α24β17-A2-ELA structure showed that three hydrogen bonds and eight vdW contacts were made between the TCR and peptide residue Ile-7 (supplemental Table S3). Thus, the reduction in binding affinity observed for α24β17 binding to the Ala-7 mutant could be attributed to a disruption of TCR-peptide contacts. Substitution of Ala for Ile at P5 in the peptide abrogated binding to undetectable levels (Table 2). The α24β17-A2-ELA structure showed that only 4 vdW contacts were made between α24β17 and the peptide residue Ile-5. Thus, the observed effect on binding affinity was surprising and was likely due to indirect effects on TCR binding.

**TABLE 2:** **Kinetic binding analysis of α24β17 to alanine-substituted peptides** NM, not measured. NB, no binding. ΔΔG° = Δ*G*° of α24β17-A2-alanine variants − Δ*G*° of α24β17-A2-ELAGIGILTV.

TCR	Peptide	Peptide sequence	Affinity -*K_D_*	Δ*G*°	ΔΔ*G*°
				*kcal/mol*	*kcal/mol*
MEL5	ELA	ELAGIGILTV	18 μm	−6.5	NM
α24β17	ELA	ELAGIGILTV	600 pm	−12.57	NM
	ELA1A	**A**LAGIGILTV	140 μm	−5.25	7.32
	ELA4A	ELA**A**IGILTV	36 μm	−6.06	6.51
	ELA5A	ELAG**A**GILTV	NB	NM	NM
	ELA6A	ELAGI**A**ILTV	41 μm	−5.98	6.59
	ELA7A	ELAGIG**A**LTV	31 μm	−6.15	6.42
	ELA8A	ELAGIGI**A**TV	21 μm	−6.38	6.19
	ELA9A	ELAGIGIL**A**V	37 μm	−6.04	6.53

Importantly, the MEL5 TCR was also very sensitive to Ala substitutions, and we observed no binding to the MEL5 TCR with Ala substituted across the ELAGIGILTV peptide (data not shown). These data demonstrate that, even though the α24β17 TCR bound with a *K_D_* of 0.6 nm, mediated by new contacts made with the MHC, the TCR was still extremely sensitive to changes in the antigenic peptide.

##### Peptide Substitutions Do Not Alter the Overall Conformation of the α24β17-A2-ELA Complex

**FIGURE 4.**
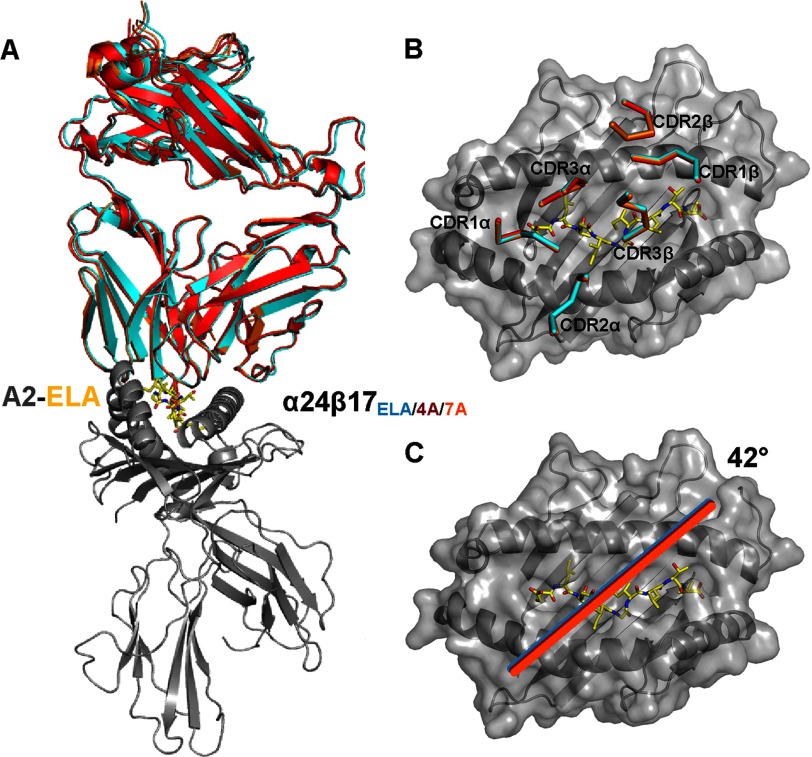
**Structural comparison of the binding mode implemented by α24β17 when interacting with A2-ELA, A2-ELA4A, and A2-ELA7A.** A2-ELA is shown in a *gray schematic*, and peptide is shown as *yellow sticks. A*, shown is the overall binding mode of α24β17 when interacting with A2-ELA (*cyan schematic*), A2-ELA4A (*red schematic*), and A2-ELA7A (*orange schematic*). α24β17 binding in a virtually identical mode to all three epitopes is shown. *B* and *C*, shown are surface representations of the A2-ELA complex (colored as in *A*) looking down at the peptide. *B*, positions of the α24β17 TCR CDR loops when interacting with A2-ELA (*cyan schematic*), A2-ELA4A (*red schematic*), and A2-ELA7A (*orange schematic*) are shown. *C*, shown is the α24β17 TCR crossing angle when interacting with A2-ELA (*cyan schematic*), A2-ELA4A (*red schematic*), and A2-ELA7A (*orange schematic*). The positions of the CDR loops and the TCR crossing angle are virtually identical across all three complexes.

As the enhanced affinity of the α24β17 TCR could largely be attributed to increased contacts between the TCR and MHC, the exquisite maintenance of peptide specificity we observed was surprising. Indeed, Ala substitutions in the peptide were predicted to have the potential to directly disrupt only a small number of TCR-pMHC contacts and were, therefore, expected to have a limited impact on binding affinity. To gain a more detailed atomic perspective on this unexpected and dramatic observation, we solved the co-complex structures of two alanine-substituted ligands where there was residual binding by the α24β17 TCR. Co-complexes of α24β17 with A2-ELAAIGILTV (A2-ELA4A) and A2-ELAGIGALTV (A2-ELA7A) enabled a detailed view of the atomic bonding between these antigens and TCR. The α24β17-A2-ELA4A and α24β17-A2-ELA7A complex structures were solved to a resolution of 2.5 and 2.7 Å, respectively. Molecular replacement was successful only in space group P4_1_ for both complexes, and the resolution was sufficiently high to show that the interface between the two molecules was well ordered and contained well defined electron density (supplemental Fig. S1). The crystallographic *R*_work_/*R*_free_ factors were 20.2 and 24.8% for α24β17-A2-ELA4A, respectively, and 20.2 and 25% for α24β17-A2-ELA7A, respectively (supplemental Table S1). Compared with the α24β17-A2-ELA structure, the overall BSA was very similar across the three co-complex structures (α24β17-A2-ELA BSA = 2705 Å^2^, α24β17-A2-ELA4A BSA = 2650.4 Å^2^, α24β17-A2-ELA7A BSA = 2781.6 Å^2^) (Table 1). Additionally, the docking geometry and the positions of the TCR CDR loops were indistinguishable between the three co-complexes (Table 1, Fig. 4). Thus, large conformational changes could not explain the large difference in binding affinity between α24β17-A2-ELA and the Ala mutants.

##### Peptide Substitutions Do Not Directly Alter Contacts between the α24β17 TCR and A2-ELA

**FIGURE 5.**
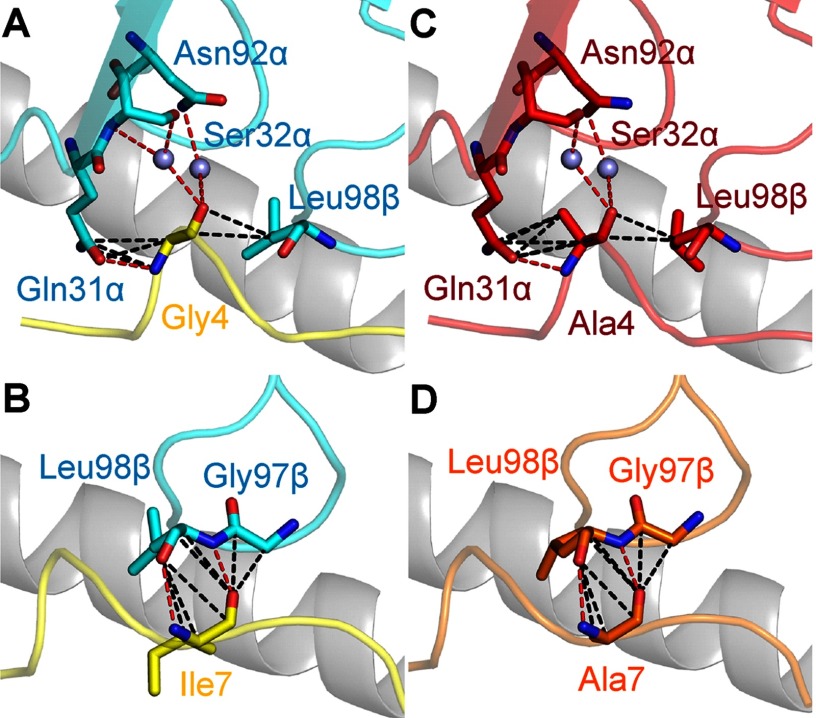
**Interactions between α24β17 and modified residues in the ELAGIGILTV peptide at positions 4 and 7.** Hydrogen bonds (<3.4 Å) are shown as *red dotted lines*, van der Waals interactions (<4.0 Å) are shown as *black dotted lines*, and water molecules are shown as *gray spheres*. Shown are α24β17 (*cyan sticks*) interactions with peptide residues (*yellow sticks*) Gly-4 (*A*) and Ile-7 (*B*). *C*, shown is α24β17 (*red sticks*) interacting with A2-ELA4A, residue Ala-4 (*red sticks*). *D*, shown is α24β17 (*orange sticks*) interacting with A2-ELA7A, residue Ala-7 (*orange sticks*). Contacts between α24β17 and the three epitopes (A2-ELA, A2-ELA4A, and A2-ELA7A) are virtually identical and cannot explain the reduced affinity between α24β17 and the modified peptides.

To further dissect the effects of the Ala substitutions on α24β17 binding, we investigated the direct consequences of these mutations on binding. The structure of α24β17 bound to A2-ELA demonstrated that peptide residue Gly-4 made four hydrogen bonds and eight vdW contacts, and Ile-7 made three hydrogen bonds and eight vdW contacts with the TCR (Fig. 5, *A* and *B*, and supplemental Table S3). The α24β17-A2-ELA4A structure revealed that the number of contacts was comparable between peptide residue Ala-4 and α24β17 (3 hydrogen bonds and 10 vdW contacts), and the α24β17-A2-ELA7A structure showed that the number of contacts was also very similar between peptide residue Ala-7 and α24β17 (two hydrogen bonds and eight vdW contacts) (Fig. 5, *C* and *D*, and supplemental Tables S4 and S5). Thus, it was unlikely that changes in contacts in the Ala peptide substitutions could directly explain the >50,000-fold difference in binding affinity between α24β17 binding to A2-ELA compared with A2-ELA4A and A2-ELA7A.

##### Peptide Substitutions Do Not Alter the Overall Conformation of Unligated A2-ELA Molecules

We next explored the possibility that Ala substitutions could impact TCR binding by altering the structure of the unligated A2-ELA molecules. We reasoned that, if α24β17 TCR binding required a conformational shift in the peptide, then more energy would be required to reach the same final bound state, thus explaining the lower binding affinity. To this end we solved the atomic structures of A2-ELA1A, A2-ELA4A, and A2-ELA8A at 2.1, 1.9, and 1.9 Å resolution, respectively (supplemental Table S6). Molecular replacement was successful in space groups P1, C121, and P12_1_1 for A2-ELA1A, A2-ELA4A, and A2-ELA8A, respectively, and the resolution was sufficiently high to show that the structures were well ordered and contained well defined electron density (supplemental Fig. S1). A comparison of these Ala mutant structures with the unligated structure A2-ELA (41) showed that each mutant did not substantially alter its structure when in complex with TCR (21, 42) (supplemental Fig. S4). These data discounted the possibility that alterations in the unligated conformation of the Ala-substituted A2-ELA peptides could explain the sensitivity of the α24β17 TCR to these single peptide mutations. Collectively, the detailed structural analysis of α24β17 TCR binding to A2-ELA and alanine substitutions thereof did not provide a clear explanation for the observed peptide specificity of α24β17 TCR binding.

##### Peptide Specificity Is Governed by Altered Entropy and a Reduction in Water Bridges

**FIGURE 6.**
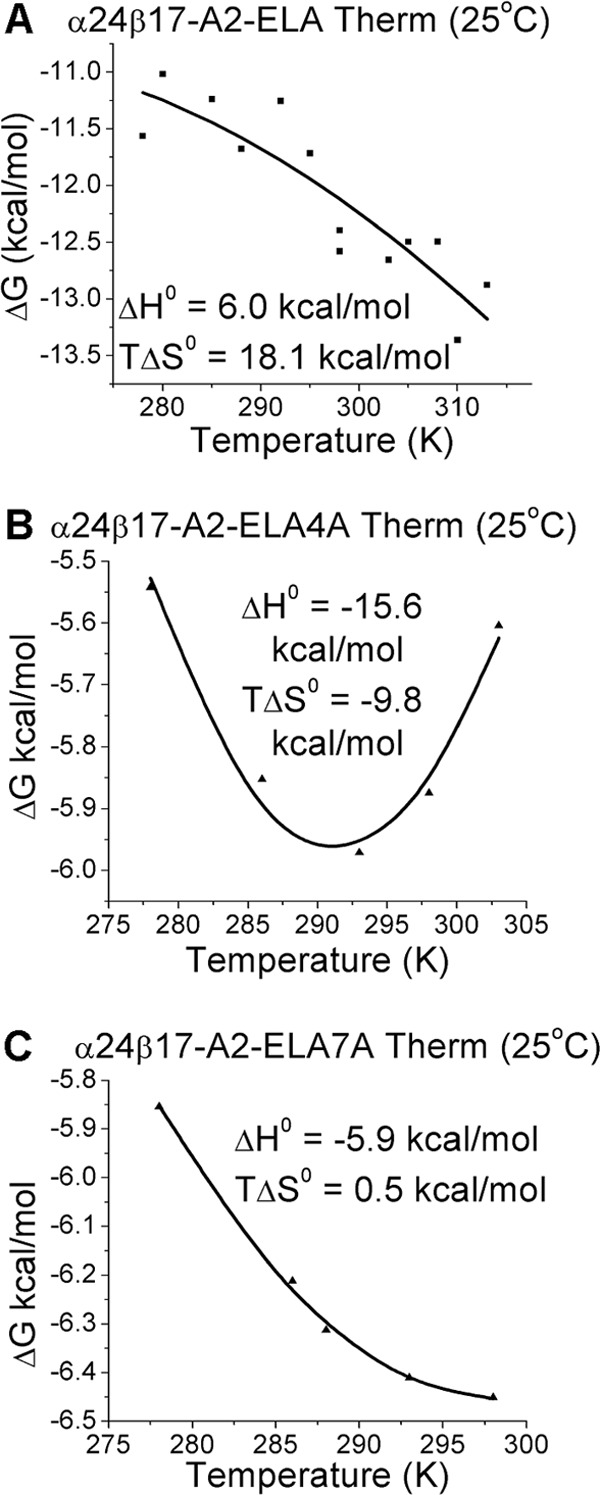
**Thermodynamic analysis of α24β17 binding to A2-ELA, A2-ELA4A, and A2-ELA7A.** The thermodynamic parameters were calculated according to the Gibbs-Helmholtz equation (Δ*G*° = Δ*H* − *T*Δ*S*°). The binding free energies, Δ*G*° (Δ*G*° = *RT*ln*K_D_*), were plotted against temperature (K) using non-linear regression to fit the three-parameter Van't Hoff equation (RTln *K_D_* = Δ*H*° − *T*Δ*S*° + ΔCp°(*T* − *T*_0_) − *T*ΔCp° ln(*T*/*T*_0_) with *T*_0_ = 298 K) as previously reported (24). Thermodynamic measurements of α24β17 binding to A2-ELA (*A*), α24β17 binding to A2-ELA4A (*B*), and α24β17 binding to A2-ELA7A (*C*) are shown. α24β17 uses a distinct thermodynamic strategy when binding to each of the three different epitopes.

We next asked to what extent the striking peptide specificity of α24β17 could be explained by thermodynamics. This was achieved by comparing the changes in enthalpy (Δ*H*°) and entropy (*T*Δ*S*°) of α24β17 binding to A2-ELA and the Ala peptide variants (Fig. 6, supplemental Figs. S5–S7). The α24β17-A2-ELA interaction (Fig. 6*A*) was entropically driven (*T*Δ*S*° ∼ 18.1 kcal/mol) and enthalpically unfavorable (Δ*H*° ∼ 6 kcal/mol), within the range observed for other TCR-pMHC interactions (from −30 to 18 kcal·mol^−1^ for Δ*H*° and from −80 to 24 kcal·mol^−1^ for *T*Δ*S*°) (43, 44). The structure of the unligated α24β17 TCR (supplemental Table S1) demonstrated that a large 8.06 Å conformational shift in CDR3β loop was required to accommodate residues toward the C terminus of the peptide (supplemental Fig. S8). Together with the observation of a favorable entropy change for binding, these observations indicated that, similar to the MEL5-A2-ELA complex (*T*Δ*S*° ∼ 8.3 kcal/mol) (21), an entropically favorable transition from order to disorder was key for driving the high affinity interaction, probably through the expulsion of ordered solvent during binding rather than a loss of order at the TCR-pMHC interface. This energetic mechanism was reversed for the α24β17-A2-ELA4A and α24β17-A2-ELA7A interactions, both binding with unfavorable entropy (*T*Δ*S*° ∼ −9.8 and −0.5 kcal/mol, respectively), and favorable enthalpy (Δ*H*° ∼ −15.6 and −5.9 kcal/mol, respectively) (Fig. 6, *B* and *C*). Thus, the loss of binding affinity observed for the interaction between the α24β17 TCR and the Ala-modified peptides seemed to be governed by a change in the order-disorder balance during binding, possibly due to differences in the interaction between the unligated molecules and solvent (44). Furthermore, although α24β17 bound to A2-ELA4A (*K_D_* = 36 μm) and A2-ELA7A (*K_D_* = 31 μm) with affinities similar to the MEL5-A2-ELA interaction (*K_D_* = 18 μm), α24β17 used a distinct thermodynamic signature (favorable enthalpy) compared with MEL5-A2-ELA (favorable entropy). Our observations that single Ala mutations cause a substantial differences in energetic binding underscore the exquisite sensitivity of this high affinity TCR to interfacial perturbations.

**FIGURE 7.**
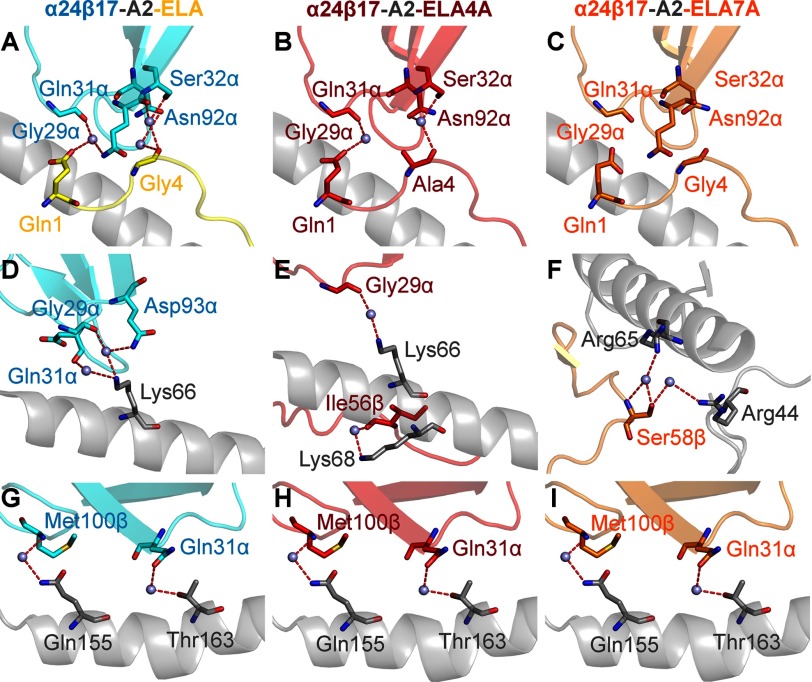
**α24β17 makes more water bridges with A2-ELA compared with A2-ELA4A and A2-ELA7A.** α24β17 binding to A2-ELA is shown in *cyan sticks*, and peptide is shown in *yellow sticks*. α24β17 binding to A2-ELA4A is shown in *red sticks*, and peptide is in *red sticks*. α24β17 binding to A2-ELA7A is shown in *orange sticks*, and peptide is in *orange sticks*. The MHC is shown in a *gray schematic*, and water molecules are shown as *gray spheres*. Hydrogen bonds (<3.4 Å) are shown as *red dotted lines*. Shown are water bridges in the α24β17-A2-ELA complex between the TCR and peptide (*A*), water bridges in the α24β17-A2-ELA4A complex between the TCR and peptide (*B*), water bridges in the α24β17-A2-ELA7A complex between the TCR and peptide (*C*), water bridges in the α24β17-A2-ELA complex between the TCR and MHCα1 helix (*D*), water bridges in the α24β17-A2-ELA4A complex between the TCR and MHCα1 helix (*E*), water bridges in the α24β17-A2-ELA7A complex between the TCR and MHCα1 helix (*F*), water bridges in the α24β17-A2-ELA complex between the TCR and MHCα2 helix (*G*), water bridges in the α24β17-A2-ELA4A complex between the TCR and MHCα2 helix (*H*), and water bridges in the α24β17-A2-ELA7A complex between the TCR and MHCα2 helix (*I*).

We then examined the role of water molecules during antigen recognition and noticed changes in the number of water bridges between the different complexes. For instance, in the α24β17-A2-ELA complex, nine water bridges were formed between the TCR and pMHC compared with only seven for α24β17-A2-ELA4A and five for α24β17-A2-ELA7A (Fig. 7). Although the reduction in water bridge-mediated hydrogen bonds could partly explain the reduction in binding affinity to the Ala-substituted peptide mutants, the difference in water bridges also suggested that the interaction between the TCR/pMHC/solvent was different in the three complexes. Here, the less favorable entropic values that were the main driving force governing the weaker binding affinity observed for the α24β17-A2-ELA4A and α24β17-A2-ELA7A interactions were most likely due to a reduction in the expulsion of ordered solvent during binding. We conclude that the reordering of solvent during antigen engagement provides the best explanation for the sensitivity of the TCR to peptide side-chain substitutions, considering the similar binding modes and the distinct thermodynamic signatures observed for the α24β17 TCR interacting with A2-ELA compared with A2-ELA4A and A2-ELA7A. Overall, these data support the idea that TCR specificity can be mediated by changes in solvent interactions between the TCR and pMHC that can occur through a “knock-on” effect due to modifications to the peptide sequence.

## DISCUSSION

The clonotypic TCR, expressed on the surface of CD8^+^ T-cells, allows recognition of peptide fragments from endogenous proteins presented at the cell surface by MHCI. TCRs discriminate between peptides and permit T-cell-mediated elimination of any cell expressing potentially dangerous intracellular proteins. Exploitation of the TCR offers exciting new possibilities for disease-specific therapies. Unlike antibodies that bind with a relatively strong affinity, TCRs bind with a weak affinity (*K_D_* = 100 nm to 270 μm) and short half-lives (0.1–12 s) (6, 7). This disparity is magnified during cancer-specific T-cell responses due to self-specific TCRs binding at the weaker end of this scale (7). The weak affinity and short half-lives of natural TCR-pMHC interactions impose severe constraints on the use of soluble TCRs for targeting cell surface-expressed pMHCs. This limitation has recently been overcome using phage display (10), yeast display (11), and computational design (12, 13) techniques that enhance TCR affinity by vastly extending the half-life of TCRs for cognate pMHC. These new developments enable cellular targeting of diseased tissue with enhanced TCR in soluble form (17).

We previously generated several high affinity TCRs directed against a range of antigens using phage display and directed evolution (5, 10, 14). Here, we investigated the interaction of one of these mutants, α24β17, derived from the MEL5 TCR that is specific for the HLA-A*0201-restricted MART-1_26–35_ antigen (45). The α24β17 TCR bound to A2-ELA with an affinity 30,000 times stronger than the MEL5 TCR (10), primarily attributed to a longer off-rate. To better understand the mechanism of high affinity TCR binding, we solved the structure of a high affinity TCR, α24β17, in complex with A2-ELA. Although α24β17 used a similar overall binding mode to MEL5 during engagement, finer examination of the structure demonstrated just three new contacts between α24β17 and the ELA peptide, compared with 41 new MHC contacts. Thus, the enhanced affinity was mediated primarily through additional interactions with the surface of the MHC molecule. This observation raised the possibility that the α24β17 TCR might exhibit reduced peptide specificity, an outcome with significant implications for the development and widespread use of high affinity TCRs. To investigate the peptide specificity of α24β17, we performed an investigation of Ala substitutions across the peptide backbone. Other investigations have shown that peptide substitutions can have a range of effects on TCR binding. Usually, substitutions in the center of the peptide have the largest effect on TCR binding, whereas substitutions at the ends of the peptide have a smaller or no effect (46–48). Surprisingly, we observed that the α24β17 TCR was highly sensitive to single Ala substitutions at all positions in the peptide with some mutations capable of completely abrogating binding.

Although the finding that α24β17 was highly sensitive to single Ala peptide mutations could be system-specific (*i.e.* may not be observed for other high affinity TCRs), it was unexpected and warranted further investigation into the molecular mechanisms underlying this effect. We solved the structure of α24β17 in complex with two Ala mutants, A2-ELA4A and A2-ELA7A. Additionally, we solved the structure of the unligated α24β17 TCR and unligated A2-ELA1A, A2-ELA4A, and A2-ELA8A molecules. Collectively, the structures demonstrated that the α24β17 used an almost identical binding strategy to engage the Ala mutants compared with the native A2-ELA and that the Ala mutations did not alter the peptide conformation of the unligated pMHCs. The total number of contacts made in the α24β17-A2-ELA complex was similar to the mutated α24β17-A2-ELA4A and α24β17-A2-ELA7A complexes and could not explain the substantially weaker binding affinity to the α24β17 TCR.

Previous analyses have shown TCRs can use a range of different thermodynamic strategies to bind to pMHC, although favorable enthalpy, most likely mediated through the formation of new bonds during ligation, is the most common driving force (43). This energetic diversity reflects the flexible binding strategies implemented by the TCR during pMHC engagement. Although conformational plasticity in the TCR CDR loops upon pMHC binding is the most common mechanism deployed (48–50), a number of studies have also shown that the TCR can remain rigid (42, 46, 51–53), enabling a “lock and key”-like interaction. A thermodynamic investigation of α24β17 binding to A2-ELA, compared with A2-ELA4A and A2-ELA7A, generated some highly unanticipated results. The α24β17 TCR used a distinct thermodynamic signature to engage A2-ELA compared with A2-ELA4A and A2-ELA7A (we observed a marked decrease in favorable entropy for α24β17 binding to A2-ELA4A and A2-ELA7A). This entropy drop suggested that differences in the ordering of solvent molecules involved during α24β17 binding could have an important role in governing antigen specificity. In support of this notion, we observed a reduction in the number of water bridges for α24β17 binding to A2-ELA4A and A2-ELA7A compared with A2-ELA. These data support the idea that peptide specificity can be mediated almost solely through changes in solvent.

In summary, we show that major improvements to TCR affinity can be gained by increasing interactions between the TCR and the MHC. Despite predictions to the contrary (15, 16), however, such an outcome need not abrogate the exquisite peptide specificity characteristic of TCR recognition. Our biophysical and thermodynamics analyses of the α24β17 TCR suggest that altered interactions with solvent molecules were the major contributor to the maintenance of peptide specificity. This observation broadens our understanding of T-cell antigen recognition and provides a new mechanism by which TCRs maintain peptide specificity.

## Supplementary Material

Supplemental Data

## Footnotes

8 The abbreviations used are:

TCR: T-cell receptor
MHCI: MHC class I
pMHC: peptide-MHC
SPR: surface plasmon resonance
Bistris propane: 1,3-bis[tris(hydroxymethyl)methylamino]propane
BSA: buried surface area
CDR: complementarity-determining region
vdW: van der Waals
TOPS: TCR/pMHC optimized protein crystallization screen.

## References

[1] Gura T. (2002) Therapeutic antibodies. Magic bullets hit the target. Nature 417, 584–586 12050630 10.1038/417584a

[2] Waldmann T. A. (2003) Immunotherapy. Past, present, and future. Nat. Med. 9, 269–277 12612576 10.1038/nm0303-269

[3] Morgan R. A. Dudley M. E. Wunderlich J. R. Hughes M. S. Yang J. C. Sherry R. M. Royal R. E. Topalian S. L. Kammula U. S. Restifo N. P. Zheng Z. Nahvi A. de Vries C. R. Rogers-Freezer L. J. Mavroukakis S. A. Rosenberg S. A. (2006) Cancer regression in patients after transfer of genetically engineered lymphocytes. Science 314, 126–129 16946036 10.1126/science.1129003 PMC2267026

[4] Porter D. L. Levine B. L. Kalos M. Bagg A. June C. H. (2011) Chimeric antigen receptor-modified T cells in chronic lymphoid leukemia. N. Engl. J. Med. 365, 725–733 21830940 10.1056/NEJMoa1103849 PMC3387277

[5] Varela-Rohena A. Molloy P. E. Dunn S. M. Li Y. Suhoski M. M. Carroll R. G. Milicic A. Mahon T. Sutton D. H. Laugel B. Moysey R. Cameron B. J. Vuidepot A. Purbhoo M. A. Cole D. K. Phillips R. E. June C. H. Jakobsen B. K. Sewell A. K. Riley J. L. (2008) Control of HIV-1 immune escape by CD8 T cells expressing enhanced T-cell receptor. Nat. Med. 14, 1390–1395 18997777 10.1038/nm.1779 PMC3008216

[6] Bridgeman J. S. Sewell A. K. Miles J. J. Price D. A. Cole D. K. (2012) Structural and biophysical determinants of αβ T-cell antigen recognition. Immunology 135, 9–18 22044041 10.1111/j.1365-2567.2011.03515.x PMC3246648

[7] Cole D. K. Pumphrey N. J. Boulter J. M. Sami M. Bell J. I. Gostick E. Price D. A. Gao G. F. Sewell A. K. Jakobsen B. K. (2007) Human TCR-binding affinity is governed by MHC class restriction. J. Immunol. 178, 5727–5734 17442956 10.4049/jimmunol.178.9.5727

[8] Aleksic M. Liddy N. Molloy P. E. Pumphrey N. Vuidepot A. Chang K. M. Jakobsen B. K. (2012) Different affinity windows for virus and cancer-specific T-cell receptors. Implications for therapeutic strategies. Eur. J. Immunol. 42, 3174–3179 22949370 10.1002/eji.201242606 PMC3776049

[9] Dunn S. M. Rizkallah P. J. Baston E. Mahon T. Cameron B. Moysey R. Gao F. Sami M. Boulter J. Li Y. Jakobsen B. K. (2006) Directed evolution of human T cell receptor CDR2 residues by phage display dramatically enhances affinity for cognate peptide-MHC without increasing apparent cross-reactivity. Protein Sci. 15, 710–721 16600963 10.1110/ps.051936406 PMC2242494

[10] Li Y. Moysey R. Molloy P. E. Vuidepot A. L. Mahon T. Baston E. Dunn S. Liddy N. Jacob J. Jakobsen B. K. Boulter J. M. (2005) Directed evolution of human T-cell receptors with picomolar affinities by phage display. Nat. Biotechnol. 23, 349–354 15723046 10.1038/nbt1070

[11] Kieke M. C. Shusta E. V. Boder E. T. Teyton L. Wittrup K. D. Kranz D. M. (1999) Selection of functional T cell receptor mutants from a yeast surface-display library. Proc. Natl. Acad. Sci. U.S.A. 96, 5651–5656 10318939 10.1073/pnas.96.10.5651 PMC21915

[12] Hawse W. F. Champion M. M. Joyce M. V. Hellman L. M. Hossain M. Ryan V. Pierce B. G. Weng Z. Baker B. M. (2012) Cutting edge. Evidence for a dynamically driven T cell signaling mechanism. J. Immunol. 188, 5819–5823 22611242 10.4049/jimmunol.1200952 PMC3375328

[13] Irving M. Zoete V. Hebeisen M. Schmid D. Baumgartner P. Guillaume P. Romero P. Speiser D. Luescher I. Rufer N. Michielin O. (2012) Interplay between T cell receptor binding kinetics and the level of cognate peptide presented by major histocompatibility complexes governs CD8^+^ T cell responsiveness. J. Biol. Chem. 287, 23068–23078 22549784 10.1074/jbc.M112.357673 PMC3391157

[14] Liddy N. Bossi G. Adams K. J. Lissina A. Mahon T. M. Hassan N. J. Gavarret J. Bianchi F. C. Pumphrey N. J. Ladell K. Gostick E. Sewell A. K. Lissin N. M. Harwood N. E. Molloy P. E. Li Y. Cameron B. J. Sami M. Baston E. E. Todorov P. T. Paston S. J. Dennis R. E. Harper J. V. Dunn S. M. Ashfield R. Johnson A. McGrath Y. Plesa G. June C. H. Kalos M. Price D. A. Vuidepot A. Williams D. D. Sutton D. H. Jakobsen B. K. (2012) Monoclonal TCR-redirected tumor cell killing. Nat. Med. 18, 980–987 22561687 10.1038/nm.2764

[15] Holler P. D. Chlewicki L. K. Kranz D. M. (2003) TCRs with high affinity for foreign pMHC show self-reactivity. Nat. Immunol. 4, 55–62 12469116 10.1038/ni863

[16] Zhao Y. Bennett A. D. Zheng Z. Wang Q. J. Robbins P. F. Yu L. Y. Li Y. Molloy P. E. Dunn S. M. Jakobsen B. K. Rosenberg S. A. Morgan R. A. (2007) High-affinity TCRs generated by phage display provide CD4^+^ T cells with the ability to recognize and kill tumor cell lines. J. Immunol. 179, 5845–5854 17947658 10.4049/jimmunol.179.9.5845 PMC2140228

[17] Sewell A. K. (2012) Why must T cells be cross-reactive? Nat. Rev. Immunol. 12, 669–677 22918468 10.1038/nri3279 PMC7097784

[18] Wooldridge L. Ekeruche-Makinde J. van den Berg H. A. Skowera A. Miles J. J. Tan M. P. Dolton G. Clement M. Llewellyn-Lacey S. Price D. A. Peakman M. Sewell A. K. (2012) A single autoimmune T cell receptor recognizes more than a million different peptides. J. Biol. Chem. 287, 1168–1177 22102287 10.1074/jbc.M111.289488 PMC3256900

[19] Wucherpfennig K. W. Allen P. M. Celada F. Cohen I. R. De Boer R. Garcia K. C. Goldstein B. Greenspan R. Hafler D. Hodgkin P. Huseby E. S. Krakauer D. C. Nemazee D. Perelson A. S. Pinilla C. Strong R. K. Sercarz E. E. (2007) Polyspecificity of T cell and B cell receptor recognition. Semin. Immunol. 19, 216–224 17398114 10.1016/j.smim.2007.02.012 PMC2034306

[20] Cole D. K. Edwards E. S. Wynn K. K. Clement M. Miles J. J. Ladell K. Ekeruche J. Gostick E. Adams K. J. Skowera A. Peakman M. Wooldridge L. Price D. A. Sewell A. K. (2010) Modification of MHC anchor residues generates heteroclitic peptides that alter TCR binding and T cell recognition. J. Immunol. 185, 2600–2610 20639478 10.4049/jimmunol.1000629 PMC3024538

[21] Cole D. K. Yuan F. Rizkallah P. J. Miles J. J. Gostick E. Price D. A. Gao G. F. Jakobsen B. K. Sewell A. K. (2009) Germ line-governed recognition of a cancer epitope by an immunodominant human T-cell receptor. J. Biol. Chem. 284, 27281–27289 19605354 10.1074/jbc.M109.022509 PMC2785656

[22] Garboczi D. N. Ghosh P. Utz U. Fan Q. R. Biddison W. E. Wiley D. C. (1996) Structure of the complex between human T-cell receptor, viral peptide, and HLA-A2. Nature 384, 134–141 8906788 10.1038/384134a0

[23] Boulter J. M. Glick M. Todorov P. T. Baston E. Sami M. Rizkallah P. Jakobsen B. K. (2003) Stable, soluble T-cell receptor molecules for crystallization and therapeutics. Protein Eng. 16, 707–711 14560057 10.1093/protein/gzg087

[24] Cole D. K. Dunn S. M. Sami M. Boulter J. M. Jakobsen B. K. Sewell A. K. (2008) T cell receptor engagement of peptide-major histocompatibility complex class I does not modify CD8 binding. Mol. Immunol. 45, 2700–2709 18243322 10.1016/j.molimm.2007.12.009

[25] Cole D. K. Rizkallah P. J. Gao F. Watson N. I. Boulter J. M. Bell J. I. Sami M. Gao G. F. Jakobsen B. K. (2006) Crystal structure of HLA-A*2402 complexed with a telomerase peptide. Eur. J. Immunol. 36, 170–179 16323248 10.1002/eji.200535424

[26] Wyer J. R. Willcox B. E. Gao G. F. Gerth U. C. Davis S. J. Bell J. I. van der Merwe P. A. Jakobsen B. K. (1999) T cell receptor and coreceptor CD8 αα bind peptide-MHC independently and with distinct kinetics. Immunity 10, 219–225 10072074 10.1016/s1074-7613(00)80022-9

[27] Cole D. K. Rizkallah P. J. Boulter J. M. Sami M. Vuidepot A. L. Glick M. Gao F. Bell J. I. Jakobsen B. K. Gao G. F. (2007) Computational design and crystal structure of an enhanced affinity mutant human CD8 αα coreceptor. Proteins 67, 65–74 17243170 10.1002/prot.21176

[28] Gostick E. Cole D. K. Hutchinson S. L. Wooldridge L. Tafuro S. Laugel B. Lissina A. Oxenius A. Boulter J. M. Price D. A. Sewell A. K. (2007) Functional and biophysical characterization of an HLA-A*6801-restricted HIV-specific T cell receptor. Eur. J. Immunol. 37, 479–486 17273992 10.1002/eji.200636243 PMC2699040

[29] Cole D. K. Gallagher K. Lemercier B. Holland C. J. Junaid S. Hindley J. P. Wynn K. K. Gostick E. Sewell A. K. Gallimore A. M. Ladell K. Price D. A. Gougeon M. L. Godkin A. (2012) Modification of the carboxy-terminal flanking region of a universal influenza epitope alters CD4^+^ T-cell repertoire selection. Nat. Commun. 3, 665 22314361 10.1038/ncomms1665 PMC3293629

[30] Karlsson R. Katsamba P. S. Nordin H. Pol E. Myszka D. G. (2006) Analyzing a kinetic titration series using affinity biosensors. Anal. Biochem. 349, 136–147 16337141 10.1016/j.ab.2005.09.034

[31] Bulek A. M. Madura F. Fuller A. Holland C. J. Schauenburg A. J. Sewell A. K. Rizkallah P. J. Cole D. K. (2012) TCR/pMHC optimized protein crystallization screen. J. Immunol. Methods 382, 203–210 22705983 10.1016/j.jim.2012.06.007 PMC3404460

[32] Winter G. (2010) Xia2: an expert system for macromolecular crystallography data reduction. J. Appl. Crystallogr. 43, 186–190

[33] Collaborative Computational Project, Number 4 (1994) The CCP4 suite. Programs for protein crystallography. Acta Crystallogr. D Biol. Crystallogr 50, 760–763 15299374 10.1107/S0907444994003112

[34] McCoy A. J. Grosse-Kunstleve R. W. Adams P. D. Winn M. D. Storoni L. C. Read R. J. (2007) Phaser crystallographic software. J. Appl. Crystallogr. 40, 658–674 19461840 10.1107/S0021889807021206 PMC2483472

[35] Emsley P. Cowtan K. (2004) Coot. Model-building tools for molecular graphics. Acta Crystallogr. D Biol. Crystallogr. 60, 2126–2132 15572765 10.1107/S0907444904019158

[36] Delano W. L. (2002) The PyMOL Molecular Graphics System, DeLano Scientific, San Carlos, CA

[37] Sami M. Rizkallah P. J. Dunn S. Molloy P. Moysey R. Vuidepot A. Baston E. Todorov P. Li Y. Gao F. Boulter J. M. Jakobsen B. K. (2007) Crystal structures of high affinity human T-cell receptors bound to peptide major histocompatibility complex reveal native diagonal binding geometry. Protein Eng. Des. Sel. 20, 397–403 17644531 10.1093/protein/gzm033

[38] Tickle I. J. Laskowski R. A. Moss D. S. (2000) Rfree and the rfree ratio. II. Calculation Of the expected values and variances of cross-validation statistics in macromolecular least-squares refinement. Acta Crystallogr. D Biol. Crystallogr. 56, 442–450 10739917 10.1107/s0907444999016868

[39] Rudolph M. G. Stanfield R. L. Wilson I. A. (2006) How TCRs bind MHCs, peptides, and coreceptors. Annu. Rev. Immunol. 24, 419–466 16551255 10.1146/annurev.immunol.23.021704.115658

[40] Borbulevych O. Y. Insaidoo F. K. Baxter T. K. Powell D. J. Jr. Johnson L. A. Restifo N. P. Baker B. M. (2007) Structures of MART-126/27–35 peptide/HLA-A2 complexes reveal a remarkable disconnect between antigen structural homology and T cell recognition. J. Mol. Biol. 372, 1123–1136 17719062 10.1016/j.jmb.2007.07.025 PMC2134917

[41] Sliz P. Michielin O. Cerottini J. C. Luescher I. Romero P. Karplus M. Wiley D. C. (2001) Crystal structures of two closely related but antigenically distinct HLA-A2/melanocyte-melanoma tumor-antigen peptide complexes. J. Immunol. 167, 3276–3284 11544315 10.4049/jimmunol.167.6.3276

[42] Borbulevych O. Y. Santhanagopolan S. M. Hossain M. Baker B. M. (2011) TCRs used in cancer gene therapy cross-react with MART-1/Melan-A tumor antigens via distinct mechanisms. J. Immunol. 187, 2453–2463 21795600 10.4049/jimmunol.1101268 PMC3166883

[43] Armstrong K. M. Insaidoo F. K. Baker B. M. (2008) Thermodynamics of T-cell receptor-peptide/MHC interactions. Progress and opportunities. J. Mol. Recognit. 21, 275–287 18496839 10.1002/jmr.896 PMC3674762

[44] Holland C. J. Rizkallah P. J. Vollers S. Calvo-Calle J. M. Madura F. Fuller A. Sewell A. K. Stern L. J. Godkin A. Cole D. K. (2012) Minimal conformational plasticity enables TCR cross-reactivity to different MHC class II heterodimers. Sci. Rep. 2, 629 22953050 10.1038/srep00629 PMC3432979

[45] Garboczi D. N. Utz U. Ghosh P. Seth A. Kim J. VanTienhoven E. A. Biddison W. E. Wiley D. C. (1996) Assembly, specific binding, and crystallization of a human TCR-αβ with an antigenic Tax peptide from human T lymphotropic virus type 1 and the class I MHC molecule HLA-A2. J. Immunol. 157, 5403–5410 8955188

[46] Bulek A. M. Cole D. K. Skowera A. Dolton G. Gras S. Madura F. Fuller A. Miles J. J. Gostick E. Price D. A. Drijfhout J. W. Knight R. R. Huang G. C. Lissin N. Molloy P. E. Wooldridge L. Jakobsen B. K. Rossjohn J. Peakman M. Rizkallah P. J. Sewell A. K. (2012) Structural basis for the killing of human beta cells by CD8^+^ T cells in type 1 diabetes. Nat. Immunol. 13, 283–289 22245737 10.1038/ni.2206 PMC3378510

[47] Miles J. J. Bulek A. M. Cole D. K. Gostick E. Schauenburg A. J. Dolton G. Venturi V. Davenport M. P. Tan M. P. Burrows S. R. Wooldridge L. Price D. A. Rizkallah P. J. Sewell A. K. (2010) Genetic and structural basis for selection of a ubiquitous T cell receptor deployed in Epstein-Barr virus infection. PLoS Pathog. 6, e1001198 21124993 10.1371/journal.ppat.1001198 PMC2987824

[48] Wu L. C. Tuot D. S. Lyons D. S. Garcia K. C. Davis M. M. (2002) Two-step binding mechanism for T-cell receptor recognition of peptide MHC. Nature 418, 552–556 12152083 10.1038/nature00920

[49] Garcia K. C. Degano M. Pease L. R. Huang M. Peterson P. A. Teyton L. Wilson I. A. (1998) Structural basis of plasticity in T cell receptor recognition of a self- peptide-MHC antigen. Science 279, 1166–1172 9469799 10.1126/science.279.5354.1166

[50] Scott D. R. Borbulevych O. Y. Piepenbrink K. H. Corcelli S. A. Baker B. M. (2011) Disparate degrees of hypervariable loop flexibility control T-cell receptor cross-reactivity, specificity, and binding mechanism. J. Mol. Biol. 414, 385–400 22019736 10.1016/j.jmb.2011.10.006 PMC3230710

[51] Chen J. L. Stewart-Jones G. Bossi G. Lissin N. M. Wooldridge L. Choi E. M. Held G. Dunbar P. R. Esnouf R. M. Sami M. Boulter J. M. Rizkallah P. Renner C. Sewell A. van der Merwe P. A. Jakobsen B. K. Griffiths G. Jones E. Y. Cerundolo V. (2005) Structural and kinetic basis for heightened immunogenicity of T cell vaccines. J. Exp. Med. 201, 1243–1255 15837811 10.1084/jem.20042323 PMC2213140

[52] Gras S. Saulquin X. Reiser J. B. Debeaupuis E. Echasserieau K. Kissenpfennig A. Legoux F. Chouquet A. Le Gorrec M. Machillot P. Neveu B. Thielens N. Malissen B. Bonneville M. Housset D. (2009) Structural bases for the affinity-driven selection of a public TCR against a dominant human cytomegalovirus epitope. J. Immunol. 183, 430–437 19542454 10.4049/jimmunol.0900556

[53] Tynan F. E. Reid H. H. Kjer-Nielsen L. Miles J. J. Wilce M. C. Kostenko L. Borg N. A. Williamson N. A. Beddoe T. Purcell A. W. Burrows S. R. McCluskey J. Rossjohn J. (2007) A T cell receptor flattens a bulged antigenic peptide presented by a major histocompatibility complex class I molecule. Nat. Immunol. 8, 268–276 17259989 10.1038/ni1432

