# Supplementary material for: T-cell Receptor Specificity Maintained by Altered Thermodynamics
Source: J Biol Chem. 2013 May 22;288(26):18766–75. doi: 10.1074/jbc.M113.464560 (PMC3696650; doi:10.1074/jbc.M113.464560)
Supplement: Supplemental Data [file supp_288_26_18766__index.html]

T-cell receptor specificity maintained by altered thermodynamics — T-cell Receptor Specificity Maintained by Altered Thermodynamics — Thermodynamic Control of TCR Specificity — Supplemental Data 

# T-cell Receptor Specificity Maintained by Altered Thermodynamics

## Supplemental Data

**Files in this Data Supplement:**

- Supplementary tables and figures (.pdf, 2.8 MB) - Supplementary tables and figures
