# Supplementary material for: T-cell Receptor Specificity Maintained by Altered Thermodynamics
Source: J Biol Chem. 2013 May 22;288(26):18766–75. doi: 10.1074/jbc.M113.464560 (PMC3696650; doi:10.1074/jbc.M113.464560)
Supplement: Supplemental Data [file supp_M113.464560_jbc.M113.464560-1.pdf]

Supplemental Data:

Specificity of an enhanced affinity T-cell receptor maintained by altered thermodynamics

Florian Madura[1*], Pierre J. Rizkallah[1*], Kim M. Miles[1], Christopher J. Holland[1], Anna M. Bulek[1], Anna Fuller[1], Andrea J.A. Schauenburg[1], John J. Miles[1,2,4], Nathanial Liddy[1,3], Malkit Sami[3], Yi Li[3], Moushumi Hossain[5], Brian M. Baker[5], Bent K. Jakobsen[3], Andrew K. Sewell[1*] and David K. Cole[1*]

[1]Cardiff University School of Medicine, Heath Park, Cardiff, CF14 4XN, UK.
[2]Human Immunity Laboratory, Queensland Institute of Medical Research, Brisbane, 4006, Australia.
[3]Immunocore Ltd., 57c Milton Park, Abingdon, OX14 4RX, UK.
[4]School of Medicine, The University of Queensland, Brisbane, 4006, Australia.
[5]Department of Chemistry and Biochemistry, University of Notre Dame, Notre Dame, Indiana 46556, USA.

Corresponding authors: Dr David Cole, E-mail: coledk@cf.ac.uk. Tel: +442920687006 and Professor Andrew Sewell, E-mail: sewellak@cf.ac.uk. Tel: +442920687055.

[*]These authors contributed equally to this study

Supplementary Table S1. Data collection and refinement statistics for unligated α24β17 TCR and complex structures

	α24β17	α24β17-A2-ELA	α24β17-A2-ELA4A	α24β17-A2-ELA7A
**PDB Code**	4JFH	4JFF	4JFD	4JFE
**Data collection**				
Space group	$P3_221$	$P4_1$	$P4_1$	$P4_1$
Cell dimensions				
a, *b*, *c* (Å)	97.14, 97.14, 123.08	121.44, 121.44, 82.3	121.49, 121.49, 82.96	121.52, 121.52, 82.15
α, β, γ (°)	90, 90, 120	90, 90, 90	90, 90, 90	90, 90, 90
Resolution (Å)	49.7-2.4 (10.7-2.4)	68.1-2.5 (10.9-2.4)	54.3-2.5 (11.0-2.5)	54.4-2.7 (11.8-2.6)
R_{merge} (%)	19.2	9.1	7.3	10.3
$I / \sigma I$	16.6	14.8	17.1	13.5
Completeness (%)	100	100	100	99.9
Redundancy	10.9	8.3	8.2	8.1
**Refinement**				
Resolution (Å)	2.4	2.4	2.5	2.7
No. reflections	25403	42931	41936	31318
R_{work} / R_{free}	20.1/24.6	21/26.3	20.2/24.8	20.2/25
No. atoms	3694	6874	6820	6765
Protein	3492	6650	6629	6667
Ligand/ion	41	65	66	32
Water	161	159	125	66
B-factors	44.63	54.73	68.38	62.91
Protein	44.60	54.74	68.91	62.91
Ligand/ion	60.79	85.12	59.51	110.62
Water	41.10	41.68	44.75	40.31
R.m.s. deviations				
Bond lengths (Å)	0.022	0.021	0.021	0.019
Bond angles (°)	1.206	1.246	1.174	1.134

* One crystal was used for solving each structure. *Values in parentheses are for highest-resolution shell.

Supplementary Table S2. Direct contacts made by the wild type, or mutant TCR residues

	TCR residue	vdW (≤4 Å)	H-bonds (≤3.4 Å)
**Wild type**	αAsp27	0	0
**MEL5 TCR**	αArg28	2 MHC	1 MHC
	αSer52	0	0
	αVal93	0	0
	αAla94	6 MHC	1 MHC
	αLys96	2 MHC	0
	βVal51	4 MHC	0
	βGly52	0	0
	βIle53	0	0
	βThr100	7 MHC	1 MHC
**Total**		**21**	**3**
**Mutant**	αPhe27	4 MHC	0
**α24β17 TCR**	αLeu28	5 MHC	0
	αArg52	3 MHC	0
	αAsp93	4 MHC	2 MHC
	αGly94	6 MHC	1 MHC
	αArg96	7 MHC	0
	βGly51	3 MHC	0
	βPro52	4 MHC	1 MHC
	βPhe53	18 MHC	1 MHC
	βMet100	2 MHC 1 ELA	1 MHC
**Total**		**68**	**6**

Supplementary Table S3: α24β17-A2-ELA contacts (residues mutated from MEL5 shown in red)

Peptide	TCR	H-bonds (≤3.2Å)	H-bonds (≤3.4Å)	vdW (≤3.5Å)	vdW (≤4Å)
Glu1$^{O\varepsilon 2/H2O}$	αGly29$^{O/H2O}$	1	1		2
Glu1^{H2O}	αGln31^{H2O}		1		2
Leu2^{O}	αGln31$^{N\varepsilon 2}$	1			1
Ala3	αGln31				2
Ala3	βLeu98			1	
Gly4^{N}	αGln31$^{O\varepsilon 1}$	1		2	3
Gly4^{H2O}	αSer32^{H2O}	1	1		
Gly4^{H2O}	αAsn92^{H2O}	1			
Gly4	βLeu98				2
Ile5	αTyr51				1
Ile5	βLeu98			2	
Ile5	βMet100				1
Gly6^{N}	βLeu98^{O}	1			
Ile7	βGly97			2	
Ile7$^{N/O}$	βLeu98$^{O/N}$	2			6
Leu8	βGly99				1
Thr9	βThr96				2
**MHC**	**TCR**	**H-bonds (≤3.2Å)**	**H-bonds (≤3.4Å)**	**vdW (≤3.5Å)**	**vdW (≤4Å)**
Glu58	αPhe27				4
Gly62	αAsp93				1
Arg65	βIle56				2
Arg65^{NH2}	αAsp93^{O}	1			3
Arg65$^{N\varepsilon}$	αGly94^{O}	1		2	3
Arg65	αArg96				7
Lys66^{H2O}	αGly29^{H2O}	1			
Lys66^{H2O}	αGly31^{H2O}		1		1
Lys66^{H2O}	αAsp93^{H2O}	1			
Lys66	αGly94				1
Ala69	βTyr49				1
Ala69	βIle56				2
Ala69	βLeu98				1
His70	βLeu98				2
Gln72	βGly51			2	1
Gln72$^{O\varepsilon 1}$	βPro52^{N}	1		1	3
Gln72$^{O\varepsilon 1}$	βPhe53^{N}	1			4
Gln72$^{N\varepsilon 2}$	βGly54^{O}		1		
Gln72	βIle56				2
Thr73	βGly97			2	
Arg75	βPhe53			2	11
Val76	βAsn30			1	
Val76	βPhe53				3
Glu154	αTyr51			1	2
Gln155	αTyr51				3
Gln155	βGly99				1
Gln155^{H2O}	βMet100^{H2O}	1			2
Arg157	αArg52			1	2
Ala158	αTyr51				2
Tyr159	αGln31			1	
Thr163^{H2O}	αGln31^{H2O}	1			2
Thr163	αLys66$^{N\zeta}$		1		
Trp167	αLeu28				3
Trp167	αGly29				2
Arg170	αLeu28			1	1

Supplementary Table S4: α24β17-A2-ELA**4A** contacts (4A peptide contacts shown in red)

Peptide	TCR	H-bonds	H-bonds	vdW	vdW
Glu1$^{O\varepsilon2/H2O}$	αGly29$^{O/H2O}$	1	1		2
Glu1$^{O\varepsilon2}$	αGln31$^{N\varepsilon2}$		1		2
Leu2^{O}	αGln31$^{N\varepsilon2}$	1			1
Ala3	αGln31				2
Ala4^{N}	αGln31$^{O\varepsilon1}$	1			6
Ala4^{H2O}	αSer32^{H2O}	1			
Ala4^{H2O}	αAsn92^{H2O}		1		
Ala4	βLeu98				2
Ile5	αTyr51				1
Ile5	βLeu98			2	
Ile5	βMet100				1
Gly6^{N}	βLeu98^{O}		1		
Ile7	βGly97			2	
Ile7$^{N/O}$	βLeu98$^{O/N}$	2			7
Thr9	βThr96			1	
**MHC**	**TCR**	**H-bonds**	**H-bonds**	**vdW**	**vdW**
Glu58	αPhe27				4
Gly62	αAsp93				1
Arg65	βIle56				2
Arg65^{NH2}	αAsp93$^{O/O\delta2}$	1	1SB		3
Arg65	αArg96				8
Lys66^{H2O}	αGly29^{H2O}	1			
Lys66	αGln31				1
Lys66	αGly94				3
Lys68^{H2O}	βIle56^{H2O}	1			
Ala69	βTyr49				1
Ala69	βLeu98				2
His70	βLeu98				1
Gln72	βGly51			2	1
Gln72$^{O\varepsilon1}$	βPro52^{N}	1		1	3
Gln72$^{O\varepsilon1}$	βPhe53^{N}	1			5
Gln72$^{N\varepsilon2}$	βGly54^{O}		1		1
Gln72	βIle56				2
Thr73	βGly97			1	1
Arg75	βPhe53			2	15
Val76	βAsn30				1
Val76	βPhe53				2
Glu154$^{O\varepsilon1/O\varepsilon2}$	αTyr51^{OH}	2			7
Gln155	αTyr51				4
Gln155	βGly99				1
Gln155^{H2O}	βMet100^{H2O}	1			2
Ala158	αTyr51				2
Tyr159	αGln31				1
Thr163^{H2O}	αGln31^{H2O}	1			2
Trp167	αLeu28				4
Trp167	αGly29				2
Arg170	αLeu28				3

Supplementary Table S5: α24β17-A2-ELA**7A** contacts (7A peptide contacts shown in red)

Peptide	TCR	H-bonds	H-bonds	vdW	vdW
Glu1$^{O\varepsilon 2}$	αGly29^{O}		1	1	1
Glu1$^{O\varepsilon 2}$	αGln31$^{N\varepsilon 2}$	1			1
Leu2^{O}	αGln31$^{N\varepsilon 2}$	1			2
Ala3	αGln31				4
Ala3	βLeu98			1	
Gly4^{N}	αGln31$^{O\varepsilon 1}$	1		2	3
Gly4	βLeu98				2
Ile5	βLeu98			1	1
Ala6^{N}	βLeu98^{O}	1			2
Ala7	βGly97				2
Ala7$^{N/O}$	βLeu98$^{O/N}$	2			6
Leu8	βThr96				1
Leu8	βLeu98				1
Leu8	βGly99				1
Thr9	βThr96			1	1
**MHC**	**TCR**	**H-bonds**	**H-bonds**	**vdW**	**vdW**
Arg44^{H2O}	βSer58^{H2O}	1			
Glu58	αPhe27				4
Gly62	αAsp93				1
Arg65	βIle56				2
Arg65^{H2O}	βSer58$^{H2O/H2O}$	2			
Arg65^{NH2}	αAsp93^{O}	1			3
Arg65$^{N\varepsilon}$	αGly94^{O}	1		1	3
Arg65	αArg96			1	6
Lys66	αGly94				2
Lys66	αLeu98				1
Lys68	βIle56				1
Ala69	βTyr49				1
Ala69	βLeu98			1	
His70	βLeu98				1
Gln72	βGly51			2	1
Gln72$^{O\varepsilon 1}$	βPro52^{N}	1		1	3
Gln72$^{O\varepsilon 1}$	βPhe53^{N}	1			8
Gln72$^{N\varepsilon 1}$	βGly54^{O}	1			4
Gln72	βIle56				2
Arg75	βPhe53			1	13
Val76	βAsn30				1
Val76	βPhe53			1	1
Glu154	αTyr51				3
Gln155	αTyr51				3
Gln155	βGly99				1
Gln155^{H2O}	βMet100^{H2O}	1			3
Ala158	αTyr51				2
Tyr159	αGln31				1
Thr163^{H2O}	αGln31^{H2O}	1			3
Trp167	αGly29				2
Arg170	αLeu28			1	1

Supplementary Table S6. Data collection and refinement statistics for A2-ELA peptide alanine substitution structures (molecular replacement).

	A2-ELA1A	A2-ELA4A	A2-ELA8A
**PDB Code**	4JFO	4JFP	4JFQ
**Data collection**			
Space group	P1	C121	$P12_11$
Cell dimensions			
a, *b*, *c* (Å)	50.28, 63.26, 75.10,	202.59, 49.11, 117.6	84.1, 58.36, 89.43
α, β, γ (°)	81.96, 76.09, 77.98	90, 90, 123	90, 109.8, 90
Resolution (Å)	45.2-2.2 (7.3-2.1)	39.7 - 2.0 (8.6-1.9)	58.6-2.0 (9.4-1.9)
R_{merge} (%)	10.0	5.2	7.0
$I / \sigma I$	18.9	10.7	16.5
Completeness (%)	78.8	98.0	97.0
Redundancy	2.1	3.5	3.4
**Refinement**			
Resolution (Å)	2.1	1.9	1.9
No. reflections	40179	70233	61271
R_{work} / R_{free}	21.8/29.6	20.5/23.9	21.9/29.6
No. atoms	6715	7089	7301
Protein	6304	6561	6378
Ligand/ion	65	101	138
Water	346	427	785
B-factors	30.33	39.41	19.30
Protein	29.96	38.79	18.12
Ligand/ion	45.03	59.33	35.14
Water	34.34	44.25	26.06
R.m.s. deviations			
Bond lengths (Å)	0.021	0.026	0.20
Bond angles (°)	1.497	1.688	1.845

* One crystal was used for solving each structure.
*Values in parentheses are for highest-resolution shell.

Supplementary Figures:

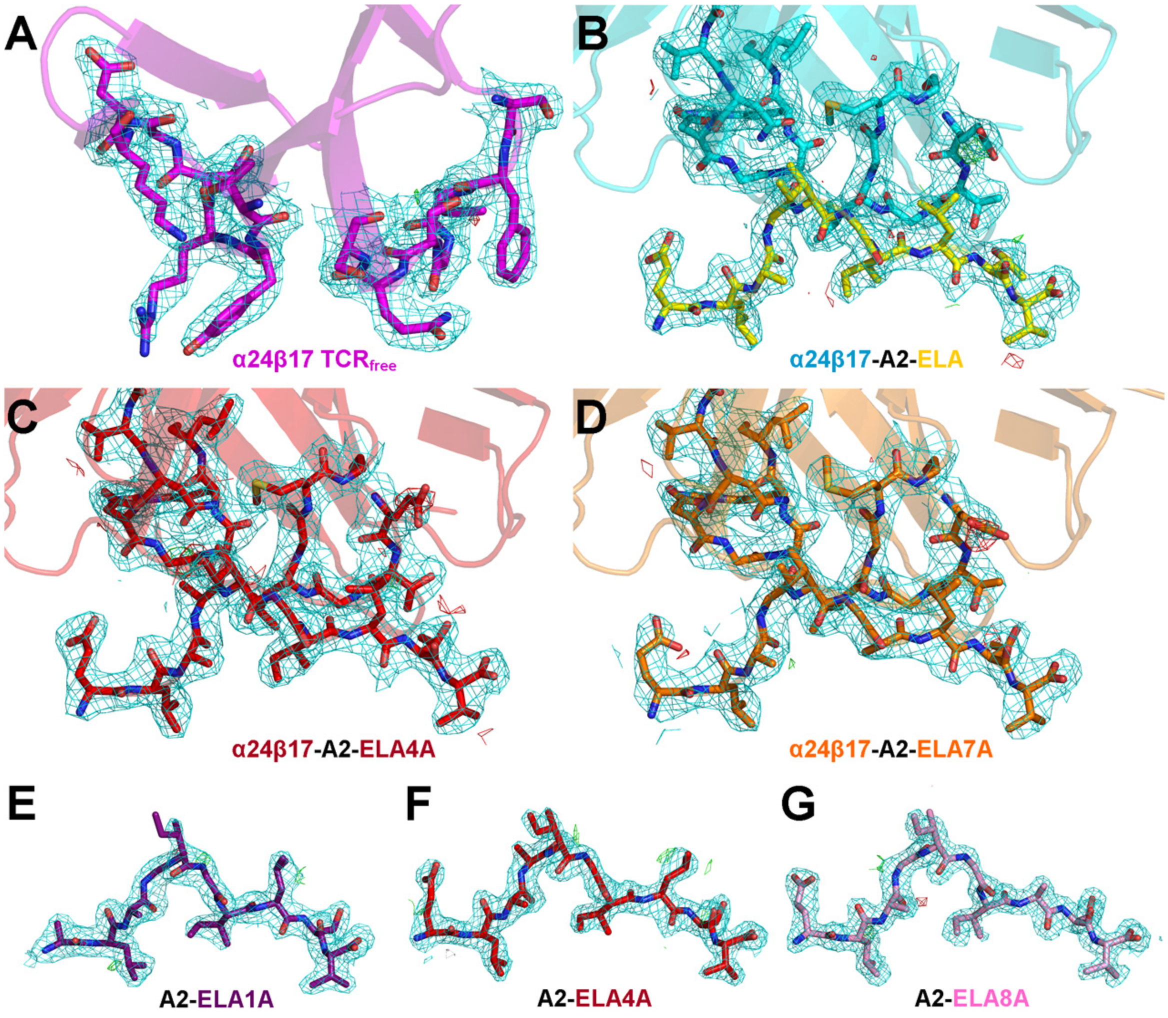

Supplementary Figure S1: 2Fo-Fc electron density maps for all structures reported

2Fo-Fc electron density maps (shown in cyan) for **(A)** α24β17 free (CDR3 loops are shown), **(B)** α24β17-A2-ELA complex (CDR3 loops and peptide are shown), **(C)** α24β17-A2-ELA4A complex (CDR3 loops and peptide are shown), **(D)** α24β17-A2-ELA7A complex (CDR3 loops and peptide are shown), **(E)** A2-ELA1A, **(F)** A2-ELA4A and **(G)** A2-ELA8A. All maps shown are within 2Å from the atoms to which they relate. Positive density is shown in green and negative density is shown in red.

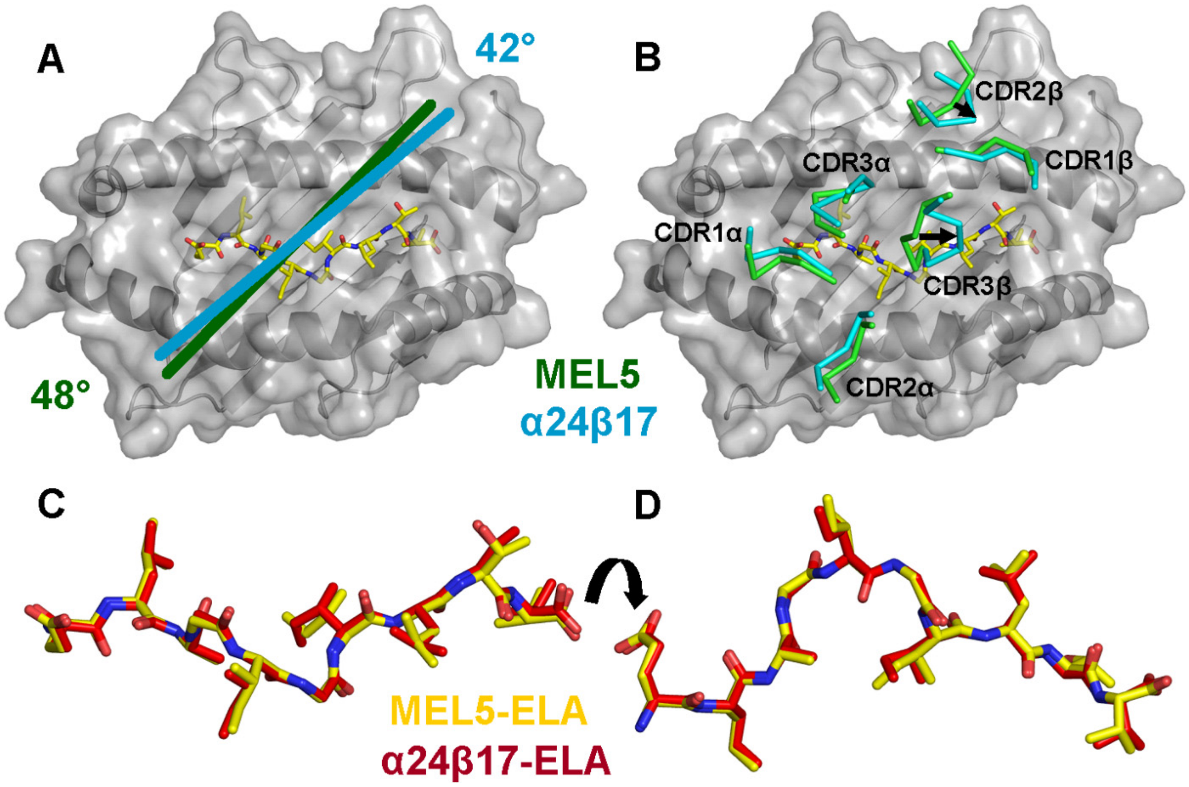

Supplementary Figure S2: Structural differences between the MEL5 *versus* α24β17 when interacting with A2-ELA.

(A) The α24β17 (cyan) and MEL5 (green) crossing angles when interacting with A2-ELA (grey surface and cartoon, peptide shown as yellow sticks). (B) Positions of the α24β17 TCR CDR loops when interacting with A2-ELA (cyan cartoon) are similar, but not identical to the positions of the MEL5 TCR CDR loops (green cartoon) when binding to A2-ELA. (C&D) The ELAGIGILTV peptide does not undergo any substantial structural changes when in complex with MEL5 (yellow) compared to α24β17 (red). (C) Top view of the MEL5 and α24β17 complexed ELA peptide. (D) Side view the MEL5 and α24β17 complexed ELA peptide.

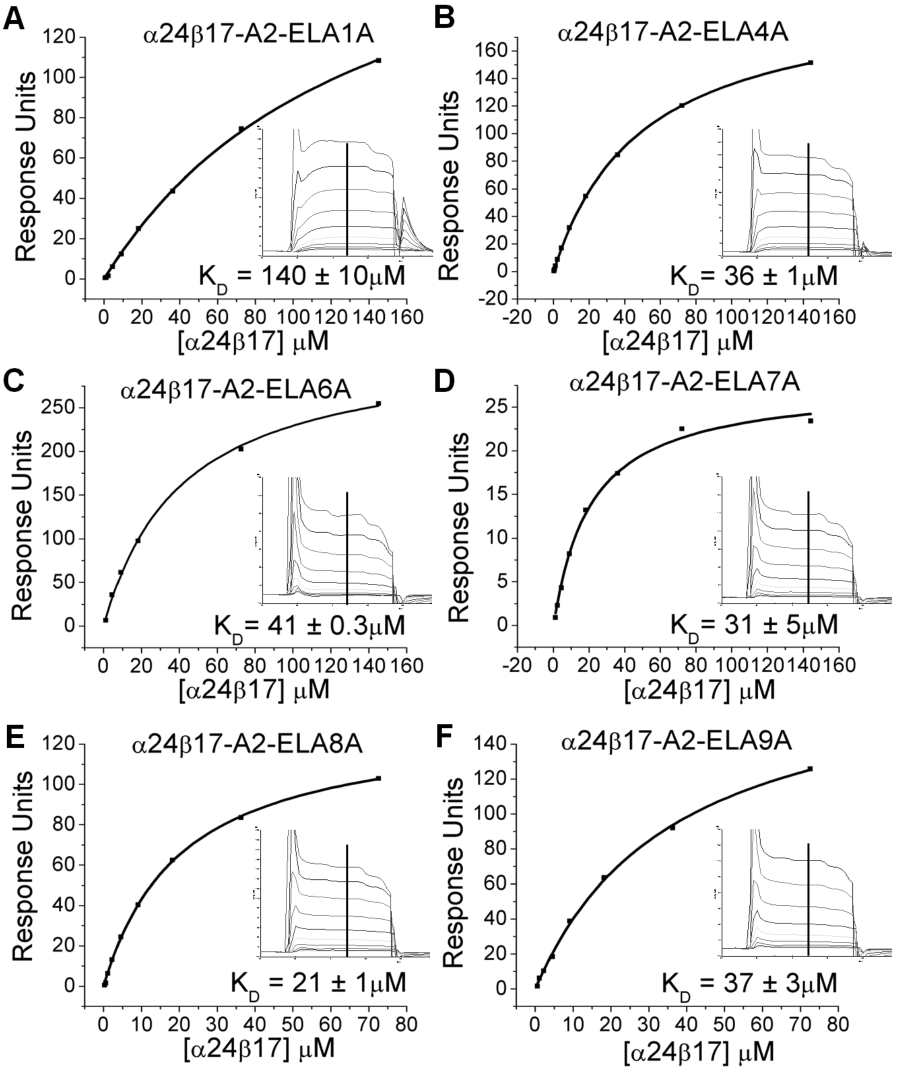

Supplementary Figure S3. α24β17 is extremely sensitive to alanine substitutions within the ELAGIGILTV peptide.

Ten serial dilutions of α24β17 were measured in four separate experiments (with different protein preparations) for each alanine substituted peptide; representative data from these experiments are plotted. The response units at each concentration of the TCR were taken from the point shown by the vertical line in each of the insets (40 seconds into the 60 second injection). Alanine substitutions in any position within the peptide reduced binding from 600pM down to wildtype like affinities (μM). The equilibrium binding constant (K_D) values were calculated using a nonlinear curve fit ($y = (P_1 x)/P_2 + x$)); mean plus SD values are shown. In order to calculate each response, α24β17 was also injected over a control sample (HLA-A*0201 in complex with ILAKFLHWL peptide, or HLA-DR1) that was deducted from the experimental data. **(A)** α24β17 binding to **A**LAGIGILTV **(B)** α24β17 binding to ELA**A**IGILTV **(C)** α24β17 binding to ELAGI**A**ILTV **(D)** α24β17 binding to ELAGIG**A**LTV **(E)** α24β17 binding to ELAGIGI**A**TV **(F)** α24β17 binding to ELAGIGIL**A**V. We observed no signal for α24β17 binding to ELAG**A**GILTV (data not shown).

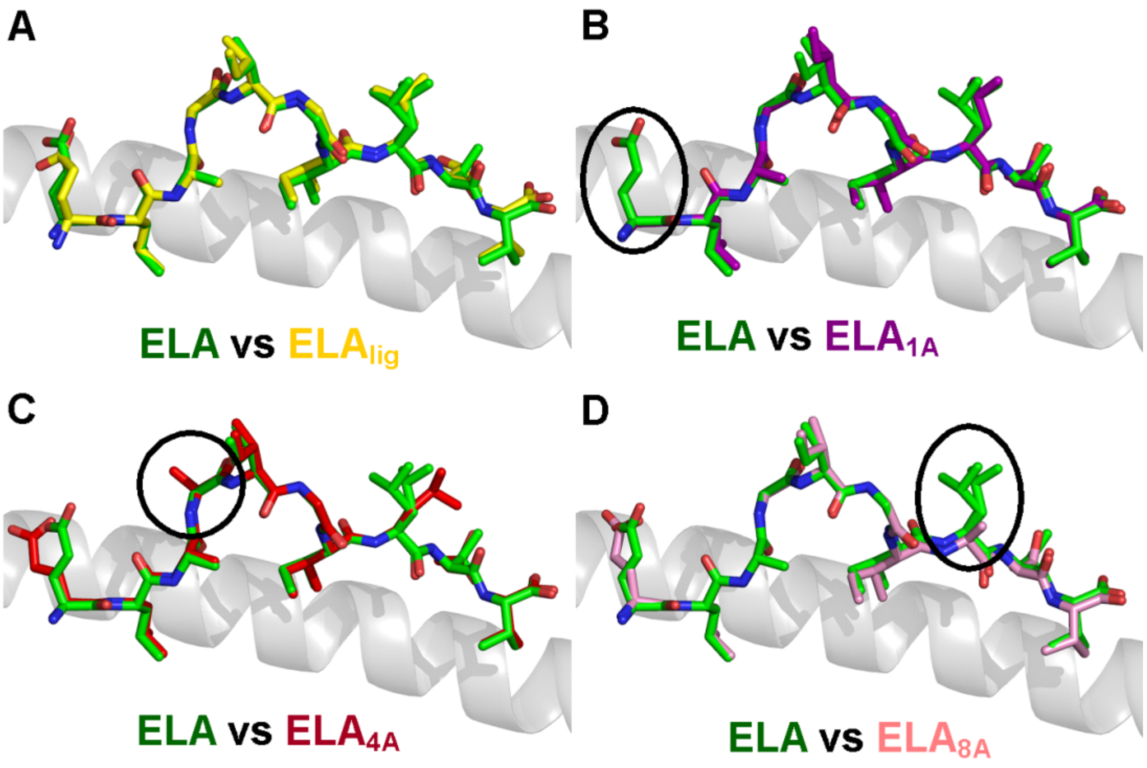

Supplementary Figure S4. Alanine substitutions do not alter the overall conformation of the unligated A2-ELA related pMHCs.

Structural comparison of the A2-ELA unligated crystal structure **(1JF1)**[1] with: **(A)** A2-ELA from the α24β17-A2-ELA complex structure, **(B)** A2-ELA1A, **(C)** A2-ELA4A and **(D)** A2-ELA8A. These structures show that the conformation of the peptide backbone is not altered by TCR binding, or by alanine substitutions. Furthermore, the positions of the solvent exposed side chains is virtually identical in all of the structures, showing that large alterations in peptide conformation in unligated state cannot account for the difference in binding affinity observed between α24β17 binding to A2-ELA compared to the alanine substituted peptides.

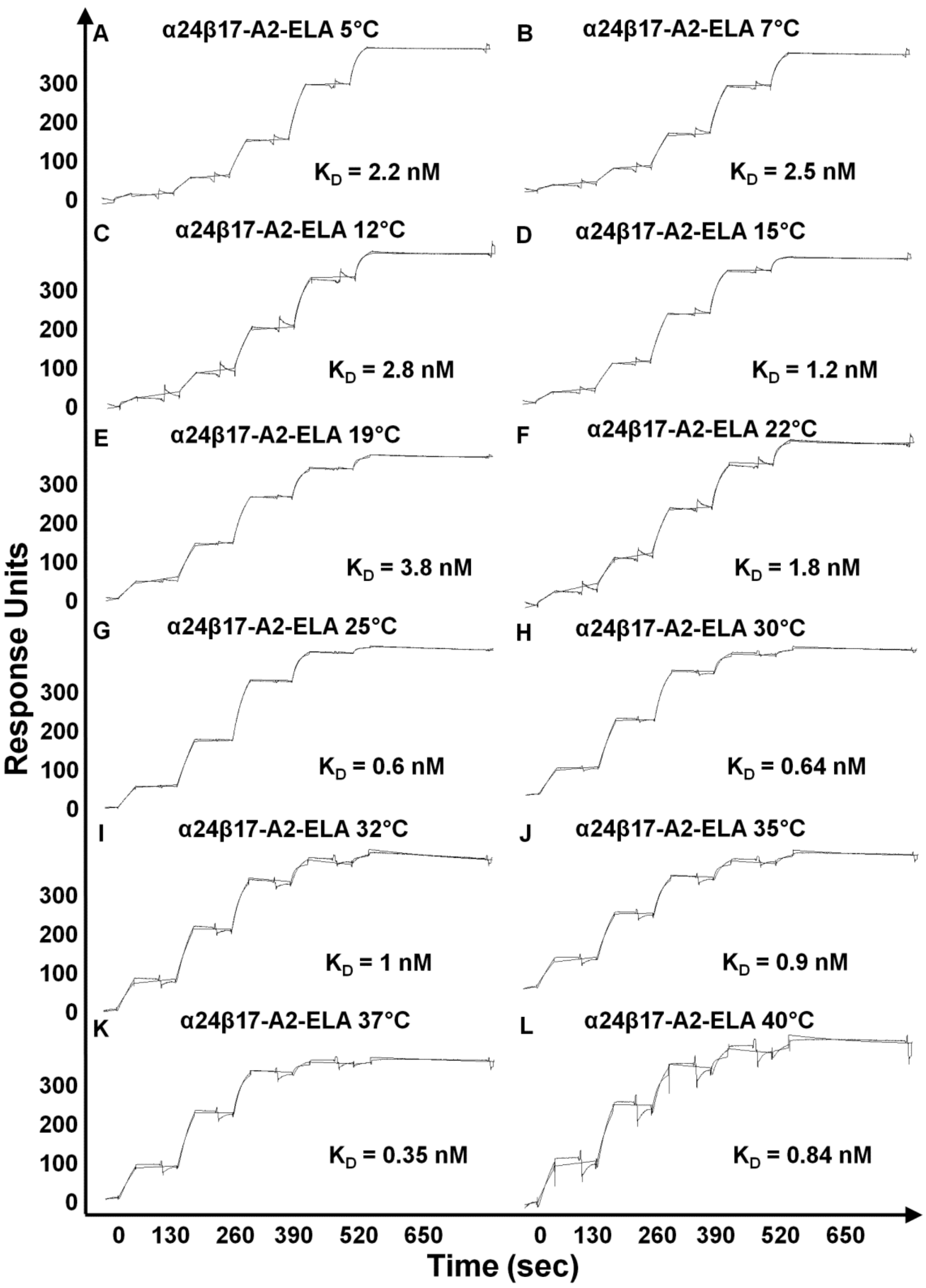

Supplementary Figure S5. Thermodynamic analysis of the α24β17-A2-ELA interaction.

Kinetic titration analysis was used to determine the affinity of the α24β17-A2-ELA interaction at: **(A)** 5°C, **(B)** 7°C, **(C)** 12°C, **(D)** 15°C, **(E)** 19°C, **(F)** 22°C, **(G)** 25°C, **(H)** 30°C, **(I)** 32°C, **(J)** 35°C, **(K)** 37°C and **(L)** 40°C. Each step in the graph indicates an injection of α24β17 using 3X increase in concentration. Thus, the five injections of α24β17 were performed at 10nM, 31nM, 94nM, 283nM, and 850nM. The raw data and the fits are shown in each panel. These data were used to fit thermodynamic parameters shown in **Figure 6.**

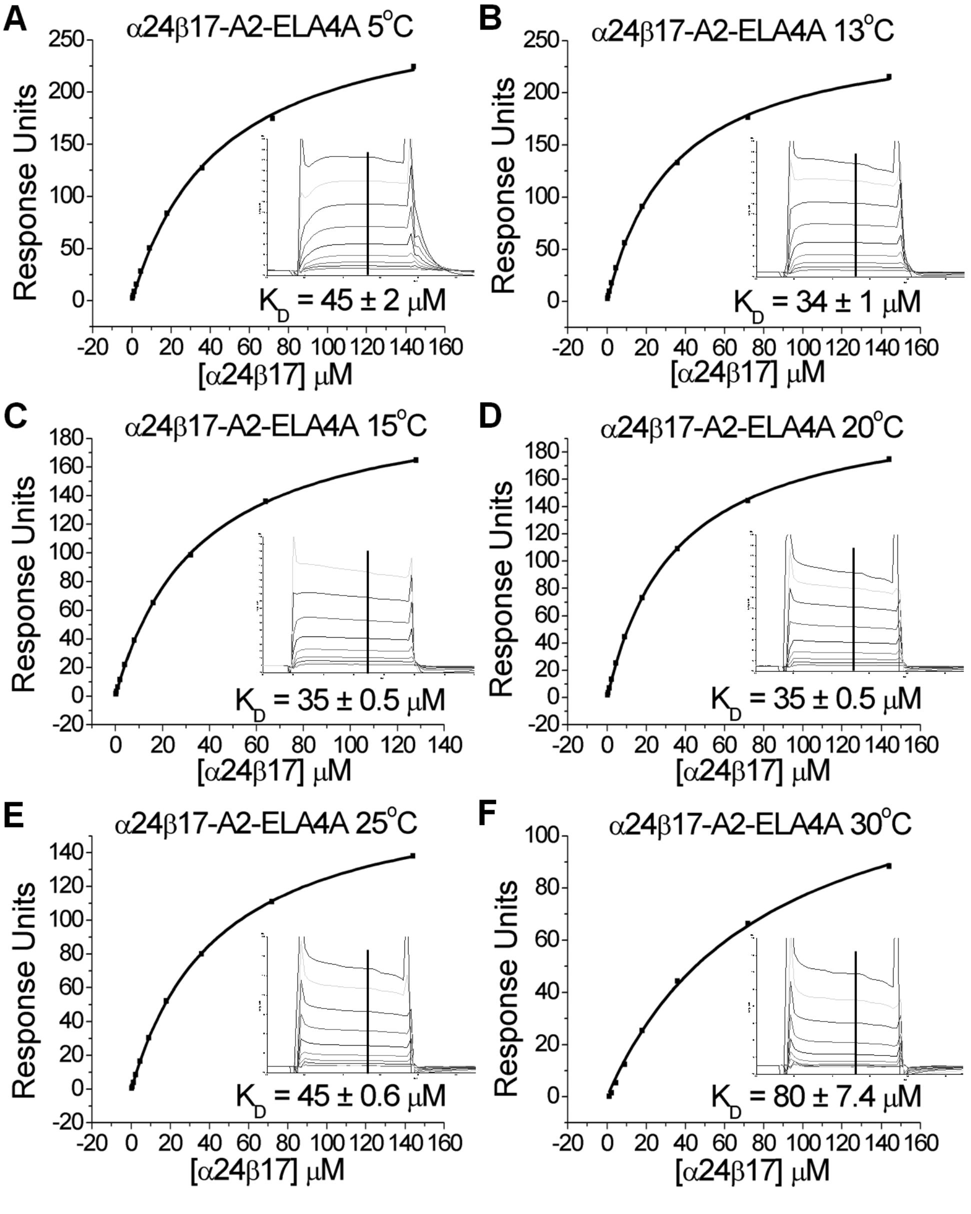

Supplementary Figure S6. Thermodynamic analysis of the α24β17-A2-ELA4A interaction.

Ten serial dilutions of α24β17 were measured in triplicate at each temperature; representative data from these experiments are plotted. The response units at each concentration of the TCR were taken from the point shown by the vertical line in each of the insets (40 seconds into the 60 second injection). The equilibrium binding constant (K_D) values were calculated using a nonlinear curve fit ($y = (P_1x)/P_2 + x)$); mean plus SD values are shown. **(A)** 5°C, **(B)** 13°C, **(C)** 15°C, **(D)** 20°C, **(E)** 25°C and **(F)** 30°C. These data were used to fit thermodynamic parameters shown in **Figure 6.**

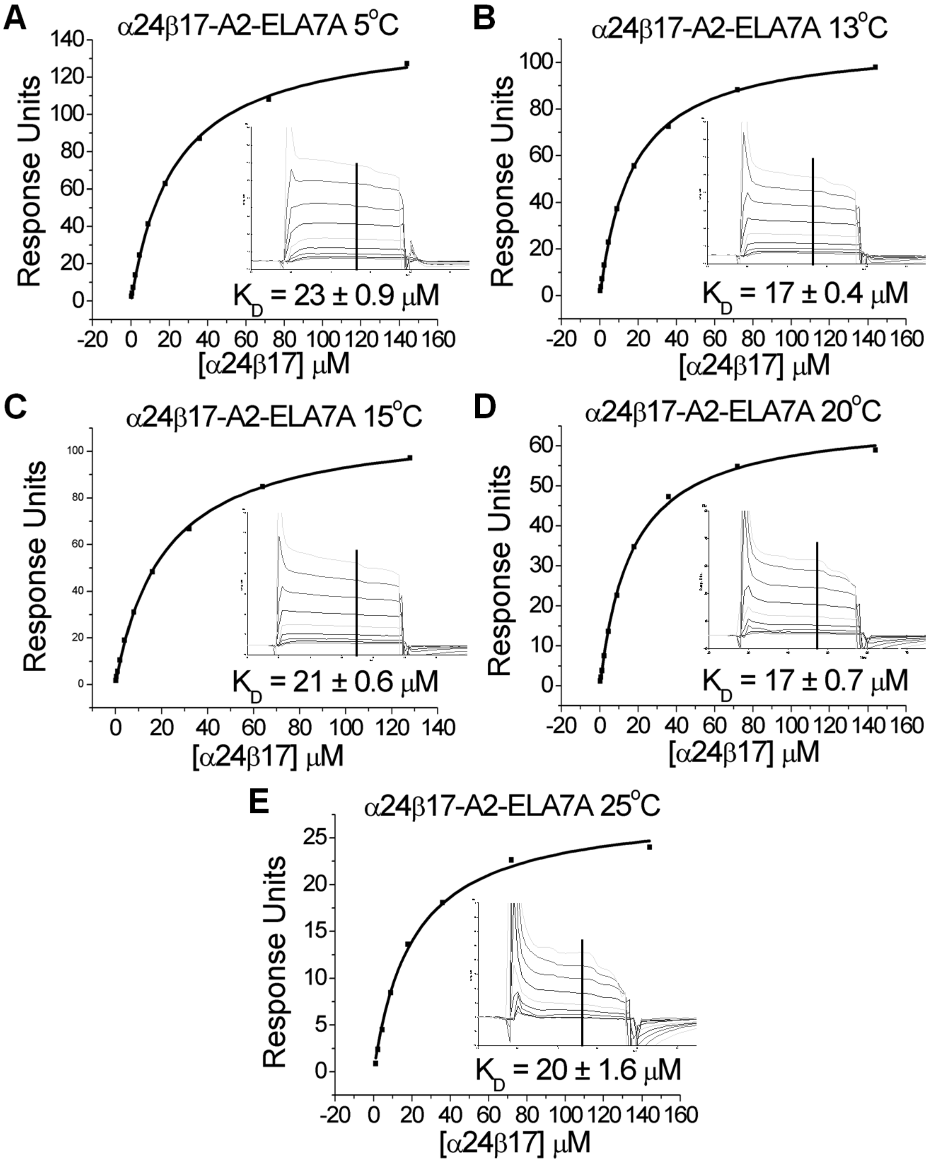

Supplementary Figure S7. Thermodynamic analysis of the α24β17-A2-ELA7A interaction.

Ten serial dilutions of α24β17 were measured in triplicate at each temperature; representative data from these experiments are plotted. The response units at each concentration of the TCR were taken from the point shown by the vertical line in each of the insets (40 seconds into the 60 second injection). The equilibrium binding constant (K_D) values were calculated using a nonlinear curve fit ($y = (P_1 x)/P_2 + x)$); mean plus SD values are shown. **(A)** 5°C, **(B)** 13°C, **(C)** 15°C, **(D)** 20°C and **(E)** 25°C. These data were used to fit thermodynamic parameters shown in **Figure 6.**

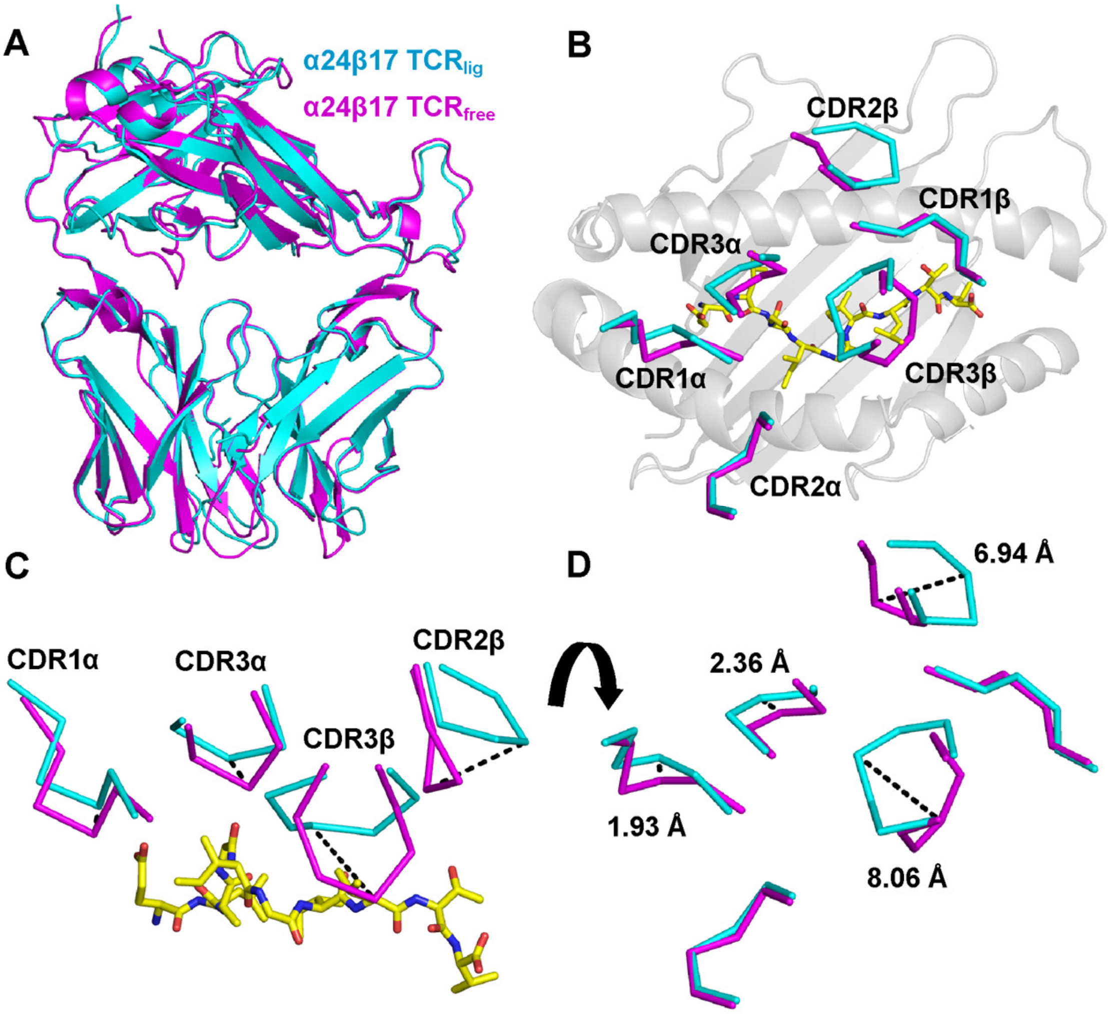

Supplementary Figure S8: α24β17 undergoes large TCR CDR movement during ligand engagement.

Comparison of the conformation of the α24β17 TCR CDR1, CDR2 and CDR3 loops in the α24β17-A2-ELA complex (α24β17$_{lig}$) *versus* α24β17 unligated (α24β17$_{free}$). **(A)** Superposition of the free (purple cartoon) and complexed (cyan cartoon) TCRs. **(B)** Superposition of the free (purple lines) and complexed (cyan lines) α24β17 TCR looking down on the peptide (yellow sticks). **(C)** Superposition of the free (purple lines) and complexed (cyan cartoon) α24β17 CDR1α, CDR3α, CDR2β and CDR3β loops from the side. The CDR3β loop has to move in order to avoid a clash with the peptide (yellow sticks) during binding. **(D)** Superposition of the free (purple lines) and complexed (cyan lines) α24β17 TCR CDR loops during binding showing backbone shifts in Å (orientation as in **(B)**). The CDR2β and CDR3β loops undergoes the largest conformational change upon binding.

References:

1. Sliz, P. et al. Crystal structures of two closely related but antigenically distinct HLA-A2/melanocyte-melanoma tumor-antigen peptide complexes. *J Immunol* **167**, 3276-84 (2001).
